# COX7A2L genetic variants determine cardiorespiratory fitness in mice and human

**DOI:** 10.1038/s42255-022-00655-0

**Published:** 2022-10-17

**Authors:** Giorgia Benegiamo, Maroun Bou Sleiman, Martin Wohlwend, Sandra Rodríguez-López, Ludger J. E. Goeminne, Pirkka-Pekka Laurila, Marie Klevjer, Minna K. Salonen, Jari Lahti, Pooja Jha, Sara Cogliati, José Antonio Enriquez, Ben M. Brumpton, Anja Bye, Johan G. Eriksson, Johan Auwerx

**Affiliations:** 1 Laboratory of Integrative Systems Physiology, École Polytechnique Fédérale de Lausanne (EPFL), Lausanne, Switzerland; 2 Finnish Institute for Health and Welfare, Helsinki, Finland; 3 Cardiac Exercise Research Group (CERG), Department of Circulation and Medical Imaging, Faculty of Medicine and Health Sciences, Norwegian University of Science and Technology (NTNU), Trondheim, Norway; 4 Department of Cardiology, St Olav’s Hospital, Trondheim, Norway; 5 Folkhälsan Research Center, Helsinki, Finland; 6 Department of Psychology and Logopedics, University of Helsinki, Helsinki, Finland; 7 Turku Institute for Advanced Studies, University of Turku, Turku, Finland; 8 Centro Nacional de Investigaciones Cardiovasculares Carlos III, Madrid, Spain; 9 Centro de Biología Molecular Severo Ochoa (CBMSO) & Institute for Molecular Biology-IUBM, Consejo Superior de Investigaciones Científicas-Universidad Autónoma de Madrid (CSIC-UAM), Madrid, Spain; 10 Instituto Universitario de Biología Molecular - IUBM (Universidad Autónoma de Madrid), Madrid, Spain; 11 Centro de Investigaciones Biomedicas en Red de Fragilidad y Envejecimiento Saludable (CIBERFES), Madrid, Spain; 12 Clinic of Medicine, St Olav’s Hospital, Trondheim University Hospital, Trondheim, Norway; 13 K.G. Jebsen Center for Genetic Epidemiology, Department of Public Health and Nursing, NTNU Norwegian University of Science and Technology, Trondheim, Norway; 14 Singapore Institute for Clinical Sciences, Agency for Science Technology and Research, Singapore, Singapore; 15 Department of Obstetrics and Gynaecology, Yong Loo Lin School of Medicine, National University of Singapore, Singapore, Singapore; 16 Department of General Practice and Primary Health Care, University of Helsinki, Helsinki, Finland

**Keywords:** Gene expression, Mechanisms of disease, Mitochondria, Energy metabolism

## Abstract

Mitochondrial respiratory complexes form superassembled structures called supercomplexes. COX7A2L is a supercomplex-specific assembly factor in mammals, although its implication for supercomplex formation and cellular metabolism remains controversial. Here we identify a role for COX7A2L for mitochondrial supercomplex formation in humans. By using human *cis*-expression quantitative trait loci data, we highlight genetic variants in the *COX7A2L* gene that affect its skeletal muscle expression specifically. The most significant *cis*-expression quantitative trait locus is a 10-bp insertion in the *COX7A2L* 3′ untranslated region that increases messenger RNA stability and expression. Human myotubes harboring this insertion have more supercomplexes and increased respiration. Notably, increased COX7A2L expression in the muscle is associated with lower body fat and improved cardiorespiratory fitness in humans. Accordingly, specific reconstitution of Cox7a2l expression in C57BL/6J mice leads to higher maximal oxygen consumption, increased lean mass and increased energy expenditure. Furthermore, Cox7a2l expression in mice is induced specifically in the muscle upon exercise. These findings elucidate the genetic basis of mitochondrial supercomplex formation and function in humans and show that COX7A2L plays an important role in cardiorespiratory fitness, which could have broad therapeutic implications in reducing cardiovascular mortality.

## Abstract (web summary)

Benegiamo et al. identify genetic variants of the mitochondrial supercomplex assembly factor COX7A2L in the skeletal muscle of mice and humans that promote cardiorespiratory fitness.

## Main

Mitochondria are indispensable organelles as they are responsible for the production of the majority of ATP in the cell. Most cellular ATP is generated by oxidative phosphorylation (OxPhos), a process through which electrons are extracted from reducing equivalents and transferred through four different respiratory complexes (RCs) present in the mitochondria inner membrane (CI–CIV). Electron transfer is coupled with the generation of a proton gradient through the mitochondrial membrane that drives the phosphorylation of ADP to ATP by the ATP-synthase complex (also known as complex V).

Although mitochondrial RCs (MRCs) are often depicted as isolated entities, it is now widely accepted that isolated complexes coexist with superassembled structures called supercomplexes (SCs)^1–3^ in the mitochondrial membrane. Within SCs, different MRCs physically interact with each other. There are different subtypes of SCs and their assembly may vary according to the organism, the metabolic state of the cell and the tissue type^4,5^; however, the main SCs in mammalian mitochondria are composed of complex I, a complex III dimer and one or more copies of complex IV^2^, whereas complex II is mostly found isolated. These SCs are also called ‘respirasomes’ because they can ‘respire’ by themselves (transfer electrons from NADH to molecular oxygen)^6^. In the current, most accepted model (the ‘plasticity model’) SCs assemble and disassemble dynamically to accommodate the cellular metabolic needs^4,5^; however, how the assembly and disassembly of SCs are regulated is not yet fully understood and their functional relevance is still debated^7^. Evidence suggests that SCs are important for organizing flux of electrons and bolster substrate channeling^8–10^, to limit the leakage of electrons and reduce reactive oxygen species (ROS) production^4,11^ and to facilitate the assembly and enhance the stability of the single complexes^12–15^. The assembly of SCs is compromised in various pathological conditions including cardiovascular diseases^16,17^, Barth syndrome^18,19^, neurological diseases^20,21^, diabetes^22^ and aging^23^. We have previously shown that SCs are induced by exercise and involved in exercise adaptation in human^24^, suggesting a possible role in muscle physiology.

COX7A2L (also known as SCAF1) is the only known SC-specific assembly factor in mammals^10^. In particular COX7A2L is required for the stable interaction of complex III with complex IV^5^, thus it plays an important role in the stabilization of the respirasome in most tissues. Previous studies, including our own, have shown that Cox7a2l promotes SC formation as well as metabolic efficiency in zebrafish and mice^25,26^. *COX7A2L* expression is induced in certain stress conditions such as hypoxia, endoplasmic reticulum (ER) and nutrient stress and its induction promotes SC formation in mammalian cells^27–29^; however, the function of COX7A2L in supercomplex formation and cellular metabolism is still controversial^30–32^.

In the present study we investigated COX7A2L function in human. We found that genetic variation significantly affects *COX7A2L* expression in several tissues with skeletal muscle showing the strongest signal. The most significant genetic variant, which is associated with increased *COX7A2L* expression specifically in muscle tissues, is a 3′ untranslated region (UTR) insertion that creates a short-repeated sequence and enhances *COX7A2L* expression by increasing messenger RNA (mRNA) stability. Human myotubes bearing this variant have increased amount of SCs and increased respiration. Notably, this highly frequent 3′ UTR variant is associated with lower body fat and improved cardiorespiratory fitness in humans. We validate these findings in C57BL/6J mice, which do not express Cox7a2l protein. Specific reconstitution of Cox7a2l expression in these mice leads to higher maximal oxygen consumption (VO_2_max), increased muscle mass and increased energy expenditure during the active phase. We further show that Cox7a2l and Cox7a2l-containing SCs are induced upon exercise specifically in the skeletal muscle. To summarize, we found that increased expression of the SC assembly factor, COX7A2L, enhances metabolic and cardiorespiratory fitness in mice and human.

## Results

### Genetic variation affects *COX7A2L* expression in human muscle

**Fig. 1:**
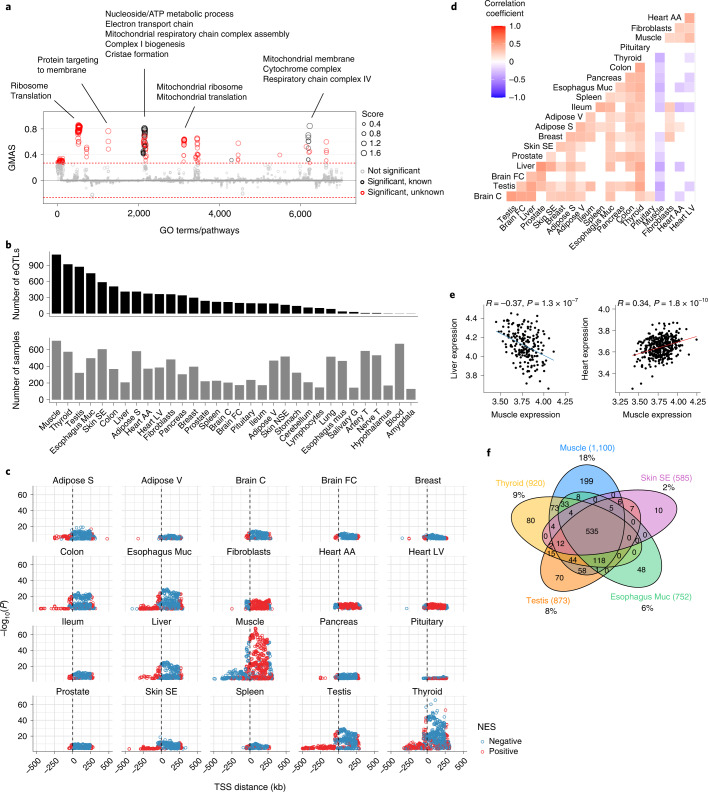
Genetic variation affects *COX7A2L* expression in human muscle. **a**, GO terms and pathways associated with *COX7A2L* expression in humans. The threshold of significant gene-pathway association is indicated by the red dashed line (Gene-Module Association Score (GMAS) >= 0.268, <=-0.268). GO terms and pathways are organized by similarity. Terms below the significance threshold are shown as gray dots. Known terms assigned to *COX7A2L* from existing annotations are shown as black dots and new terms above the threshold are shown in red. Dot size represents the GMAS (data from https://systems-genetics.org^33^). **b**, Number of significant *COX7A2L cis*-eQTLs per tissue (top) and number of samples per tissue (bottom) in the GTEx database. Tissue abbreviations are listed in Supplementary Table 3. **c**, *COX7A2L cis*-eQTLs by tissue plotted according to their distances from the *COX7A2L* TSS (*x* axis). Each dot represents one *cis*-eQTL and dot colors represent the effects of the alternative alleles on *COX7A2L* expression (NES, normalized effect size; blue, negative; red, positive). Only the 20 tissues with the highest number of *COX7A2L cis*-eQTLs are shown. **d**, Pearson pairwise correlations between tissues based on *COX7A2L* expression. Non-significant correlations (*P* > 0.05) are shown as blank squares. Only the 20 tissues with the highest number of *COX7A2L cis*-eQTLs are shown. **e**, Correlation of *COX7A2L* normalized expression in the muscle with expression in the liver (left) or heart (right), two-sided Pearson *r* and *P* value are shown above the graphs. **f**, *COX7A2L cis*-eQTLs overlap among the five tissues with the highest number of *cis*-eQTLs; the total numbers of *cis*-eQTLs per tissue are shown in parenthesis, percentages indicate the proportion of tissue-specific *cis*-eQTLs. Data source, GTEx database (https://www.gtexportal.org) v.8 release (**b**-**f**).

**Extended Data Fig. 1:**
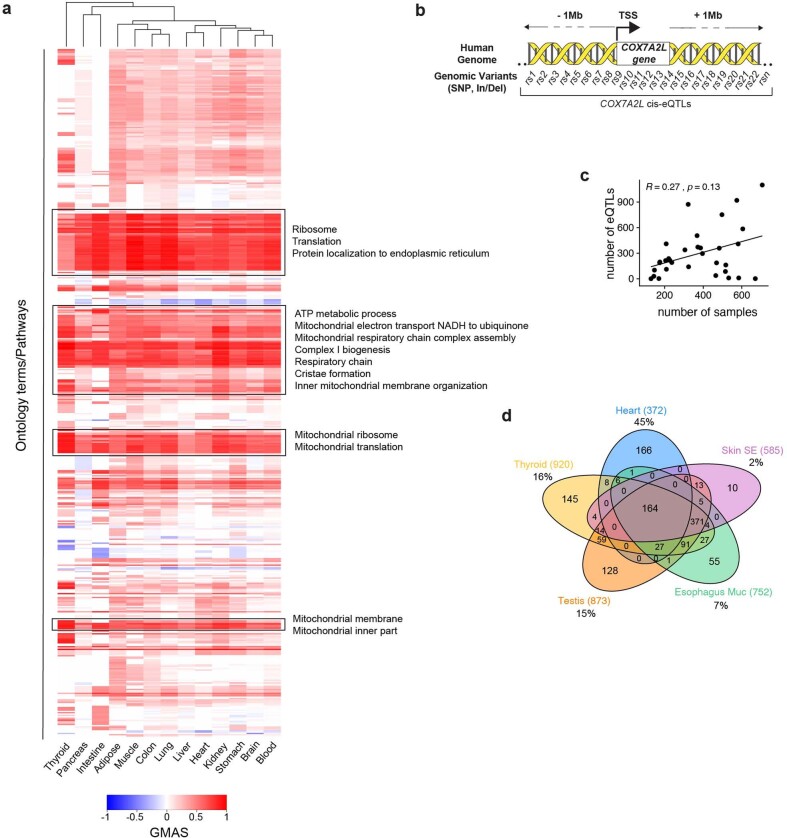
Genetic variation affects *COX7A2L* expression in human muscle. **a**. Heatmap of ontology terms and pathways associated with *COX7A2L* expression in different human tissues. All terms significantly associated with *COX7A2L* expression in at least one tissue (GMAS >=0.268, <=-0.268) are shown. Ontology terms and tissues are organized by similarity. Boxed terms are indicating representative ontology term with high correlation with *COX7A2L*. Data from https://systems-genetics.org^33^. **b**. Schematic representation of the GTEx *cis*-eQTL analysis for the *COX7A2L* gene. Variants located within ±1 Mb from the *COX7A2L* gene and affecting its expression were considered *cis*-eQTLs. **c**. Correlation between the number of samples per tissue and the number of significant *COX7A2L cis*-eQTLs per tissue. Two-sided Spearman r and p value are shown above the graph. **d**. *COX7A2L cis*-eQTLs overlap among 5 representative tissues; next to the tissue name, the total number of *cis*-eQTLs is shown in parenthesis, percentages indicate the proportion of tissue-specific *cis*-eQTLs.

To gain insights into *COX7A2L* function in humans we used the GeneBridge analysis platform (https://systems-genetics.org)^33^. GeneBridge integrates 1,337 datasets with over 265,000 human samples from different tissues and allows to interrogate a gene’s function through its correlation or anticorrelation with other genes for which the Gene Ontology (GO) terms and biological pathway annotations are already known. We found that *COX7A2L* expression correlates positively with genes belonging to many mitochondria-related ontology terms and pathways (Fig. 1a), including several that were previously unknown, such as complex I biogenesis, cristae formation, mitochondrial translation and respiratory chain complex IV (Fig. 1a and Supplementary Table 1). These associations were conserved across different human tissues (Extended Data Fig. 1a and Supplementary Table 1).

Given this strong association with mitochondrial function, we asked whether genetic variation affects *COX7A2L* expression and thus SC formation, in humans. We used the publicly available Genotype-Tissue Expression (GTEx) database v.8 release, which provides whole-genome sequencing and RNA expression data of 54 tissues across approximately 1,000 individuals^34^ (http://www.gtexportal.org/home/). We looked at expression quantitative trait loci (eQTLs) located in proximity (±1 Mb) of the *COX7A2L* transcription start site (TSS) (*cis*-eQTLs; Extended Data Fig. 1b). Considering an estimated average number of *cis*-eQTLs per gene of ~180 in GTEx^35^, we found a high number (1,427) of significant *cis*-eQTLs for *COX7A2L* (false discovery rate (FDR) < 0.05, *P* < 2.2 × 10^-4^) in 32 different tissues (Fig. 1b and Supplementary Table 2; tissue full names and abbreviations used thereafter are described in Supplementary Table 3). We observed a large variation in the number of *COX7A2L cis*-eQTLs per tissue (range 1–1,100; Fig. 1b). As sample size significantly affects *cis*-eQTL mapping^35,36^, we tested whether the number of *COX7A2L cis*-eQTLs per tissue was dependent on the sample size. No significant correlation between the number of *COX7A2L cis*-eQTLs per tissue and the number of samples per tissue was observed (Extended Data Fig. 1c), suggesting tissue-specific regulation of *COX7A2L* expression. Notably, skeletal muscle harbors the highest number (1,100) and the most significant *cis*-eQTLs (Fig. 1b,c). Furthermore, muscle tissues (skeletal muscle and heart) are distinct from most other tissues in the effect of the *cis*-eQTLs alternative allele on *COX7A2L* expression; the majority of the *cis*-eQTLs downstream of the TSS are associated with opposite changes in *COX7A2L* expression in muscle versus most other tissues (Fig. 1c). Indeed, when looking at how the 20 tissues with the highest number of *COX7A2L cis*-eQTLs correlate on the basis of *COX7A2L* expression levels, the muscle correlates positively with the heart and negatively with most other tissues such as thyroid, testis, spleen, brain, skin, the intestinal tract (esophagus, ileum and colon) and liver (Fig. 1d,e). To assess tissue-specific regulation of *COX7A2L* expression, we looked at the proportion of *cis*-eQTLs that overlap in the top five tissues (muscle, thyroid, testis, esophagus mucosa and skin sun exposed). Skeletal muscle has double the amount of specific *cis*-eQTLs (18%) compared to the thyroid (9%) and testis (8%) and almost ten times more compared to the skin (2%) (Fig. 1f). A similarly higher proportion of tissue-specific *cis*-eQTLs was observed in the heart (Extended Data Fig. 1d), suggesting that regulation of *COX7A2L* expression is specific for muscle tissues. Altogether, our analysis of *COX7A2L cis*-eQTLs in the GTEx database revealed that several gene variants in human affect *COX7A2L* expression in different tissues; the skeletal muscle has a unique *cis*-eQTL profile and the regulation of *COX7A2L* expression is muscle tissue-specific.

### *COX7A2L* lead *cis*-eQTL affects mRNA stability

**Fig. 2:**
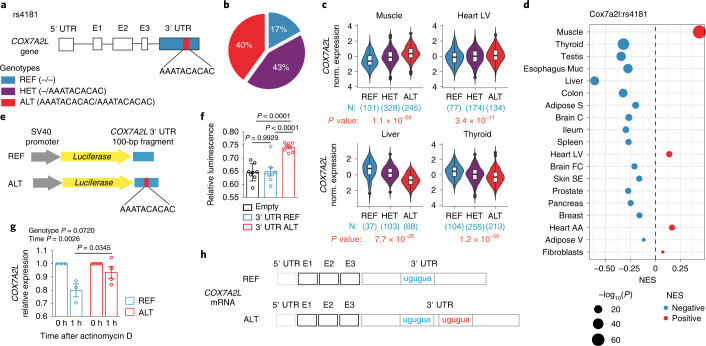
*COX7A2L* lead *cis*-eQTL affects mRNA stability. **a**, Schematic representation of rs4181 location and genotypes. **b**, rs4181 genotype frequency in all populations combined (data from 1000 Genomes Project phase 3)^37^. **c**, Normalized expression of *COX7A2L* in four representative tissues. Data are from the GTEx database, samples are grouped by genotype. The number of samples per tissue per genotype and the *cis*-eQTL *P* values are indicated. The statistical analysis used to determine the *P* value is described in https://gtexportal.org. **d**, NES of rs4181 ALT allele on *COX7A2L* expression in different tissues in the GTEx database. Dot color represents the directionality of the effect (blue, negative; red, positive) and dot size is proportional to the *P* value. The statistical analysis used to determine the *P* value is described in https://gtexportal.org. All tissues for which rs4181 is a significant *cis*-eQTL are shown. Tissues are ordered from bottom to top by *P* value. **e**, Schematic representation of the pGL3-promoter luciferase vectors used in **f**. **f**, Relative luminescence of HEK-293T cells transfected with pGL3-promoter-empty, pGL3-promoter-3′ UTR-REF or pGL3-promoter-3′ UTR-ALT. Cells were co-transfected with pRL-CMV vector and firefly luminescence was normalized to Renilla luminescence to correct for transfection efficiency (empty *n* = 8, 3′ UTR-REF *n* = 8, 3′ UTR-ALT *n* = 8). **g**, *COX7A2L* expression in seven differentiated myotubes lines (REF, *n* = 3; ALT, *n* = 4) before and 1 h after inhibition of transcription with actinomycin D (10 μg ml^−1^). **h**, Schematic representation of *COX7A2L* mRNA REF and ALT alleles. Exons (E1–E3) and 5′ UTR and 3′ UTR are depicted with the repeat present only in the ALT allele shown in red. Data are represented as violin plots and Tukey’s box and whiskers plots (superimposed) (**c**). Box plots center represents the median, lower and upper hinges represent the first and third quartile (25th and 75th percentiles). The whiskers extend to the largest and smallest value up to 1.5 × IQR from the hinge. IQR, interquartile range. Data points beyond the whiskers are plotted individually. Data are represented as mean ± s.e.m. (**f**,**g**). Statistical analysis was conducted by one-way analysis of variance (ANOVA) (**f**) and two-way ANOVA and Sidak’s multiple comparisons test (**g**). Source data

**Extended Data Fig. 2:**
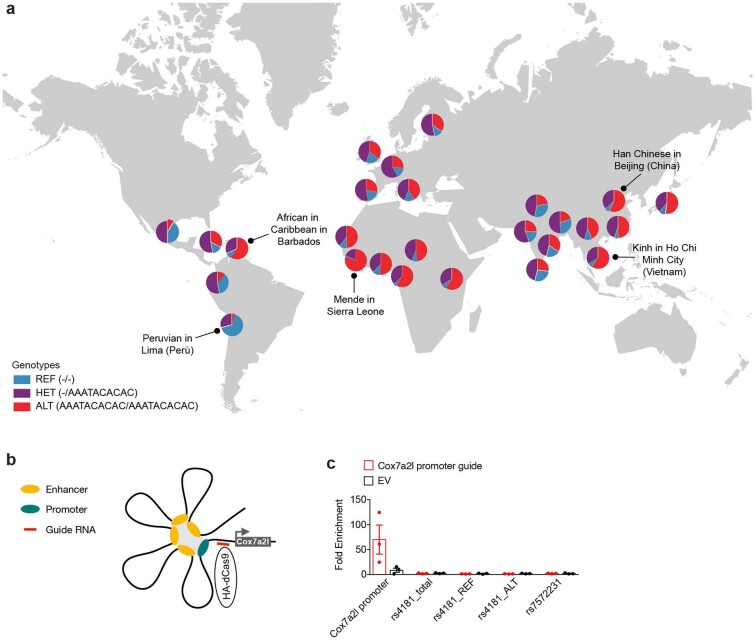
*COX7A2L* lead *cis*-eQTL is a 3’UTR insertion that increases mRNA stability. **a**. rs4181 genotype frequency by population (data from 1000 Genomes Project Phase 3). See also Supplementary Table 4. **b**. Schematic illustration of the dCAS9-HA *COX7A2L* promoter tagging experiment. **c**. Fold enrichment compared to IgG control measured by ChIP-qPCR of the *COX7A2L* promoter, rs4181_total (primers amplifying both REF and ALT 3’UTR alleles), rs4181_REF (primers amplifying only the REF 3’UTR allele), rs4181_ALT (primers amplifying only the REF 3’UTR allele), or rs7572231, another *COX7A2L cis*-eQTL located far upstream from rs4181 (n = 3; red = cells transfected with *COX7A2L* promoter guide vector, black = cells transfected with empty vector (EV)). Data are represented as mean ± SEM. Source data

To investigate the mechanism of genetic variation in *COX7A2L* expression in humans, we focused on the most significant *cis*-eQTL in the GTEx database (rs4181). Throughout this manuscript, we use the term ‘lead’ *cis*-eQTL to indicate the *cis*-eQTL that has the smallest association *P* value with *COX7A2L* expression or variants in linkage disequilibrium (LD) with the lead *cis*-eQTL. Notably, the lead *COX7A2L cis*-eQTL is located within the gene. Specifically, this variant corresponds to an insertion of 10 bp in the 3′ UTR of *COX7A2L* (Fig. 2a). The possible genotypes are −/− (reference allele in homozygosis; hereafter REF), −/AAATACACAC (heterozygous; hereafter HET) and AAATACACAC/AAATACACAC (alternative allele in homozygosis; hereafter ALT) (Fig. 2a). This variant is very frequent in the population with a minor allele frequency (MAF) of 0.383 in the 1000 Genomes Project database^37^ (Supplementary Table 4). Similar MAF is observed in the gnomAD database^38^ (MAF = 0.393; Supplementary Table 4). The REF allele represents the minor allele in the general population with most individuals being either heterozygous or homozygous for the ALT allele (83%; Fig. 2b). To note, the frequency of this allele is not equal across populations: 80% of the individuals are homozygous for the ALT allele in the Mende population in Sierra Leone, whereas only 3% of the population has this genotype among the Peruvian in Lima^37^ (Extended Data Fig. 2a and Supplementary Table 4), which suggests that one of the two alleles may be favored in certain environmental conditions. We found that rs4181 is a significant *cis*-eQTL in 19 different tissues in the GTEx database and has the most robust effect on expression in the muscle and thyroid (*P* *=* 1.1 × 10^−68^ and 1.2 × 10^−50^, respectively) (Fig. 2c,d). Consistent with our previous observation, the effect of this variant on *COX7A2L* expression is different according to the tissue: the association of the 10-bp insertion with *COX7A2L* expression is positive in the muscle, heart and cultured fibroblasts but negative in all other tissues (Fig. 2c,d). This finding again highlights the tissue-specific regulation of *COX7A2L* expression in humans. To test whether the rs4181 variant is sufficient to modulate expression, we cloned a portion of *COX7A2L* 3′ UTR with or without the 10-bp insertion in a luciferase reporter vector (Fig. 2e). An increase in luminescence was observed in HEK-293T cells transfected with the luciferase reporter harboring the 3′ UTR with the 10-bp insertion as in carriers of the ALT allele (Fig. 2f).

To dissect the molecular mechanism by which the 3′ UTR insertion increases expression in cultured cells, we first explored whether the 3′ UTR insertion is a transcriptional enhancer (whether this region increases expression by looping to the COX7A2L promoter). First, we genotyped HEK-293T cells and confirmed that this line is heterozygous for the rs4181 variant (has both REF and ALT alleles; Methods). We then took advantage of the CRISPR-Cas9 technology and co-transfected HEK-293T cells with a guide RNA targeting the *COX7A2L* promoter and a hemagglutinin (HA)-tagged dCas9 as described^39^ (Extended Data Fig. 2b). We then performed chromatin immunoprecipitation using an HA-specific antibody and measured by qPCR the enrichment of *COX7A2L* promoter and the 3′ UTR region containing the rs4181 variant. We also tested another genomic region containing the *cis*-eQTL rs7572231 located ~20 kb upstream of rs4181. While we were able to immunoprecipitate and enrich for the *COX7A2L* promoter, no co-enrichment for the two alleles of rs4181 (REF and ALT) or rs7572231 genomic regions was observed (Extended Data Fig. 2c). We thus concluded that the 3′ UTR region of the COX7A2L gene does not loop to the promoter and may not act by increasing transcription itself.

The 3′ UTR regions harbor sequences that can be bound by RNA-binding proteins and modulate RNA levels by affecting transcript stability. To test this hypothesis, we obtained primary myoblasts from seven homozygous individuals (REF, *n* = 3; ALT, *n* = 4). These human myoblasts were cultured for 10 d in differentiation medium to induce myotubes differentiation, after which transcription was blocked with actinomycin D and RNA collected after 0 and 1 h. Myotubes homozygous for the REF allele had lower *COX7A2L* mRNA levels after actinomycin D treatment, suggesting increased mRNA degradation (Fig. 2g). To note, the 10-bp insertion creates a short-repeated sequence in the RNA 3′ UTR (Fig. 2h). Short-repeated sequences separated by 1–10 nucleotides are preferred by several RNA-binding proteins (RBPs) and might favor binding of RBPs harboring multiple RNA recognition motifs or cooperative binding of RBP dimers^40^.

Taken together, these results demonstrated that *COX7A2L* lead *cis*-eQTL is a 3′ UTR insertion that is sufficient to increase gene expression in vitro by increasing mRNA stability.

### COX7A2L lead *cis*-eQTL affects cellular respiration

**Fig. 3:**
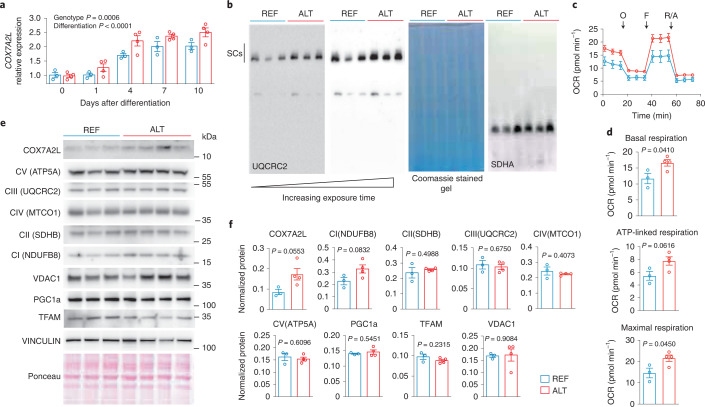
*COX7A2L* lead *cis*-eQTL affects cellular respiration. **a**, *COX7A2L* mRNA expression in human myoblasts after 0, 1, 4, 7 or 10 d of differentiation (REF, *n* = 3; ALT, *n* = 4). **b**, BN–PAGE immunoblot with UQCRC2 immunostaining (left panels) in isolated mitochondria from differentiated human myotubes cultured in glucose-free medium for 48 h. Coomassie stained gel and complex II (SDHA) immunostaining are shown on the right as loading controls. Each sample is an independent biological replicate. The experiment was repeated twice with similar results. **c**, Oxygen consumption rate in differentiated human myotubes measured during a mitochondrial stress test using a Seahorse flux analyzer. Cells were differentiated and then grown in glucose-free medium for 48 h. O, oligomycin; F, FCCP (carbonyl cyanide-p-trifluoromethoxyphenylhydrazone); R/A, rotenone/antimycin (REF, *n* = 3; ALT *n* = 4). **d**, Average basal, ATP-linked and maximal respiration calculated from **c**. OCR, oxygen consumption rate. **e**, Western blot analysis of differentiated human myotube lines cultured for 48 h in glucose-free medium. **f**, Western blot quantification of **e** (REF, *n* = 3 independent biological replicates; ALT, *n* = 4 independent biological replicates). Data are represented as mean ± s.e.m. Each dot and each sample used for BN–PAGE and western blot represent a different myoblast line. Statistical analysis conducted was two-way ANOVA (**a**) and two-sided Student’s *t*-test (**d**,**f**). Source data

**Extended Data Fig. 3:**
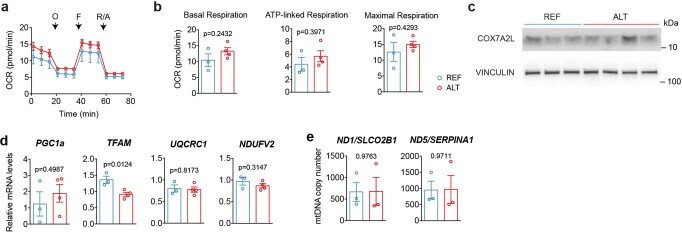
Myotubes harbouring the 3’UTR insertion have increased SCs and increased respiration. **a**. Oxygen consumption rate in human myoblasts measured during a mitochondrial stress test using a Seahorse flux analyser. Cells were grown in high-glucose medium (O = Oligomycin, F = FCCP, R/A = Rotenone/Antimycin) (REF n = 3, ALT n = 4). **b**. Average basal, ATP-linked and maximal respiration calculated from **a**. **c**. Western blot analysis of COX7A2L protein expression in human myotube lines. **d**. mRNA expression of the indicated genes measured by qRT–PCR in human myotube lines. **e**. mtDNA copy number in human myotube lines. For **a-c** myoblasts were differentiated for 7 day and then cultured for 48 h in high glucose medium. For **d-e** myoblasts were differentiated for 7 day and then cultured for 48 h in glucose-free medium. Data are represented as mean ± SEM. Statistical analysis for b: two-sided student’s t-test. Source data

Next, we assessed the metabolic phenotype of human myoblasts harboring the REF and ALT allele in homozygosis. We first confirmed that differentiated myotubes homozygous for the ALT allele have higher expression of *COX7A2L* (Fig. 3a). To note, the difference in *COX7A2L* expression between genotypes becomes only apparent in myotubes, suggesting that it is specific for differentiated muscle cells (Fig. 3a). We then asked whether differences in *COX7A2L* expression in human myotubes translate in differences in SC formation. To address this, we extracted mitochondria from differentiated human myotubes cultured in glucose-free medium (10 mM galactose) for 48 h and performed blue native (BN)–PAGE. As expected, human myotubes homozygous for the ALT allele had increased SCs (Fig. 3b). We then tested whether increased *COX7A2L* expression and increased supercomplex formation affects cellular respiration. For this, we measured respiration by Seahorse flux analyzer (Agilent) in differentiated myotubes cultured in glucose-free medium for 48 h. Myotubes homozygous for the ALT allele had higher basal, ATP-linked and maximal respiration (Fig. 3c,d). No significant difference in cellular respiration was observed when cells were cultured in high-glucose medium (25 mM glucose) (Extended Data Fig. 3a,b). Consistent with these results, myotubes homozygous for the ALT allele had higher COX7A2L protein expression, compared to myotubes homozygous for the REF allele, when cultured in glucose-free medium (Fig. 3f), whereas no differences were observed when cells were cultured in high glucose (Extended Data Fig. 3c). Cells in glucose-free medium are forced to rely on mitochondrial respiration to produce ATP and this has been linked to increased COX7A2L expression and increased MRC rearrangement into SCs^27^. To rule out increased mitochondrial biogenesis driving the observed differences in the ALT allele myotubes, we measured other MRC subunits as well as mitochondrial biogenesis markers both at the protein and mRNA levels in the two genotypes (Fig. 3e,f and Extended Data Fig. 3d) and found no significant differences. Additionally, we measured mitochondrial DNA copy number as a proxy of mitochondrial content in the REF and ALT allele myotubes and found no significant differences (Extended Data Fig. 3e).

These data show that differentiated myotubes with higher COX7A2L expression have higher SC formation and are able to adapt better to nutrient deprivation by increasing mitochondrial respiration.

### COX7A2L lead *cis*-eQTL affects cardiorespiratory fitness

**Fig. 4:**
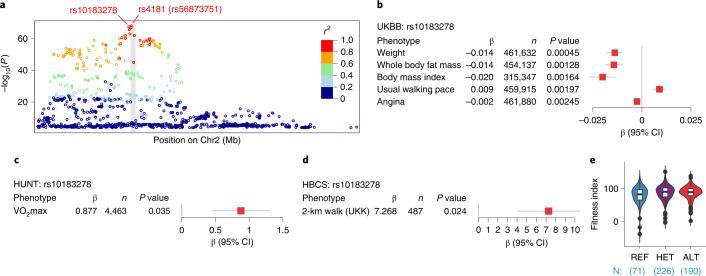
*COX7A2L* lead *cis*-eQTL affects cardiorespiratory fitness. **a**, Regional plot of the *COX7A2L* genomic region. *COX7A2L* muscle *cis*-eQTLs within 1 Mb of the TSS are plotted according to their position on Chr2 (*x* axis) and their *P* value (*y* axis). Variants are color-coded according to their LD (*r*^2^) with the lead *COX7A2L cis*-eQTL variant rs4181. The gray vertical shade indicates the location of *COX7A2L* gene. **b**, Mendelian randomization analysis on the second-most significant *COX7A2L* muscle *cis*-eQTL (rs10183278) and metabolic and fitness-related phenotypes in the UKBB. Representative phenotypes are shown (Supplementary Table 5). **c**, Association between the second-most significant *COX7A2L cis*-eQTL (rs10183278) with VO_2_max measured in the HUNT3 study. **d**, Mendelian randomization analysis between the second-most significant *COX7A2L cis*-eQTL (rs10183278) and the 2-km walk test (UKK) in the HBCS (Supplementary Table 5). **e**, Fitness index of the HBCS participants. Individuals are grouped by genotype. The number of participants per genotype is indicated. Data are represented as mean ± s.e. (**b**–**d**) and violin plots and Tukey’s box and whiskers plots (superimposed) (**e**). Box plots center represents the median, lower and upper hinges represent the first and third quartile (25th and 75th percentiles). The whiskers extend to the largest and smallest value up to 1.5 × IQR from the hinge. Data points beyond the whiskers are plotted individually. Statistical analysis used to determine the *P* value is described in Methods (**b**–**d**).

We next tested whether *COX7A2L* 3′ UTR insertion is associated with metabolic and cardiorespiratory fitness-related phenotypes in human cohorts. We first looked into the IEU GWAS database (https://gwas.mrcieu.ac.uk/datasets/)^41^, which contains summary statistics from, among others, the UK Biobank (UKBB), which is a biobank that contains genotype data, as well as a wide variety of phenotypic data for about 500,000 individuals^42^. We performed two-sample Mendelian randomization, which is a method that allows testing for causal effects of gene expression on phenotypes through a genetic variant^43,44^. The lead *cis*-eQTL for *COX7A2L* in GTEx, rs4181 (previously annotated as rs56873751), is a small insertion that is not present in most GWAS studies. For this reason, we investigated the second-most significant *cis*-eQTL, rs10183278, a single-nucleotide polymorphism (SNP) that is in high LD with rs4181 (*r*^2^ = 0.952; Fig. 4a). rs10183278 was significantly associated with several metabolic phenotypes (FDR < 0.05, *P* < 0.02; Supplementary Table 5 and Fig. 4b). Notably, an increase in *COX7A2L* expression was associated with reduced body weight, reduced body fat and body mass index (BMI) and increased usual walking pace (Fig. 4b). The UKBB has only a limited number of phenotypes that allow assessing cardiorespiratory fitness and muscle function. Examples include walking pace^45^, an indicator of overall physical fitness and grip strength, which is more specific for upper body strength. To validate the role of *COX7A2L* in overall cardiorespiratory fitness, we hence analyzed participants from the Trøndelag Health (HUNT) study. The HUNT study is a large population-based cohort study that includes questionnaire data, clinical measurements and biological samples from more than 120,000 individuals^46^. In a sub-study of HUNT (HUNT3 fitness study^47,48^), maximal oxygen uptake (VO_2_max) was measured in 4,631 individuals. VO_2_max measurement is the gold standard for assessing cardiorespiratory fitness^49^. Again, as the most significant skeletal muscle *cis*-eQTL for *COX7A2L* in GTEx, rs4181, is not present in the HUNT cohort, we investigated the second-most significant *cis*-eQTL, rs10183278 and showed it to be significantly associated with VO_2_max (Fig. 4c). To further validate these findings, we also analyzed participants from an additional independent cohort, the Helsinki Birth Cohort Study (HBCS). In this cohort several tests were performed to assess physical fitness in 1,078 individuals aged 67–77 years^50^, of which 606 took part in the Urho Kaleva Kekkonen (UKK) 2-km walk test. This test evaluates cardiorespiratory fitness without the requirements for maximal physical efforts^51,52^ and is a valid substitute for maximal physical efforts tests (VO_2_max) in elderly individuals and patients with obesity^53,54^. Mendelian randomization analysis of rs10183278 indicated that increased *COX7A2L* expression in the muscle was associated with several HBCS phenotypes (FDR < 0.2; Supplementary Table 5) with UKK showing the biggest effect (effect size of 7.27 test score units *COX7A2L* normalized effect size, *P* = 0.02; Fig. 4d and Supplementary Table 5). Specifically, individuals with higher *COX7A2L* expression in the muscle (alternative allele) have 11% higher fitness index (average fitness index 78.7 ± 28 (REF), 87.4 ± 23 (HET) and 87.4 ± 20 (ALT); Fig. 4e). To summarize, using three independent human cohorts, we showed that *COX7A2L* lead *cis*-eQTL is associated with metabolic and cardiorespiratory fitness-related phenotypes.

### A Cox7a2l mutation affects cardiorespiratory fitness in mice

**Extended Data Fig. 4:**
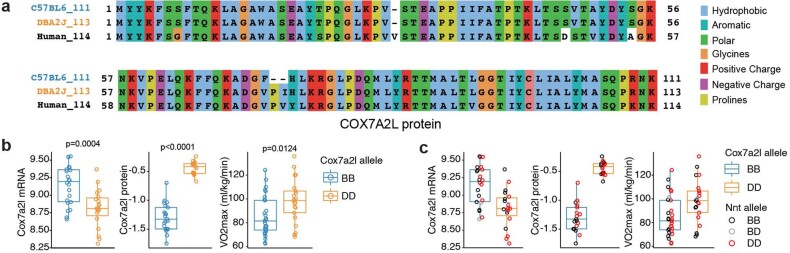
A spontaneous *Cox7a2l* mutation affects cardiorespiratory fitness in mice. **a**. Alignment of COX7A2L/Cox7a2l protein sequence between C57BL/6 J mice (111aa), DBA/2 J (113aa) mice and human (114aa). **b**. Left and middle panel: *Cox7a2l* RNA and protein levels measured in the liver of 41 (RNA) and 38 (protein) BXD strains on chow diet. Right panel: VO_2_max measured after training in 50 BXD strains on high-fat diet. Each dot is a different strain and represents the average of n = 3-5 mice. Strains are grouped based on their *Cox7a2l* allele (BB = C57BL/6 J, short allele; DD = DBA/2 J, long allele). N = pooled 3-5 mice per strain. **c**. Same data as in b, grouped based on their *Cox7a2l* allele, dot color represents the Nnt allele. In b and c data are represented as box and whiskers, box plots center represents the median, lower and upper hinges represent the first and third quartile (25^th^ and 75^th^ percentiles). The whiskers extend to the largest and smallest value up to 1.5*IQR from the hinge (IQR = inter-quartile range). Statistical analysis for **b**: two-sided student’s t-test. Data from GeneNetwork (https://www.genenetwork.org). Source data

Human genetic analyses have the limitation that several confounding factors, including environmental factors or other genetic variants co-inherited with the variant of interest, may affect the observed association of an SNP with an outcome. Environmental factors are difficult to control and correct for in human but can be tightly controlled in mice. To determine whether specific changes in Cox7a2l expression are associated with metabolic and cardiorespiratory fitness-related phenotypes, we took advantage of the presence of a known *Cox7a2l* genetic polymorphism in common mouse inbred strains. C57BL/6J mice have a six-nucleotide deletion in the *Cox7a2l* gene that truncates the protein by two amino acids and renders it unstable^10^ and incapable to interact with complex IV^5^. DBA/2J mice, as well as most other mouse strains and humans have the long, stable *Cox7a2l* form (Extended Data Fig. 4a). We thus looked into the BXD mouse genetic reference population; a large panel of well-characterized recombinant inbred strains derived from crosses between C57BL/6J and DBA/2J mice^26,55–57^ (www.genenetwork.org). This population segregates for the *Cox7a2l* polymorphism. Specifically, the BXD strains bearing the C57BL/6J allele for *Cox7a2l* (BB allele) have higher basal levels of *Cox7a2l* mRNA, compared to the strains bearing the DBA/2J allele (DD allele), likely due to a compensatory mechanism (Extended Data Fig. 4b, left), but as expected have lower levels of Cox7a2l protein^5^ (Extended Data Fig. 4b, middle). In agreement with the original observation in C57BL/6J mice^10^, we previously showed that BXD strains carrying the *Cox7a2l* BB allele lack some of the SCs and that this trait maps to a QTL containing the *Cox7a2l* gene^26^. Here we observed that, on a high-fat diet, strains with the DD allele (long *Cox7a2l* form) have higher VO_2_max after training (Extended Data Fig. 4b, right). No difference in VO_2_max between mice carrying the BB and DD allele was observed in the BXD population at baseline or on chow diet.

C57BL/6J mice are also homozygous for a loss-of-function mutation in the nicotinamide nucleotide transhydrogenase (Nnt) gene^58^ that renders them more susceptible to metabolic dysfunctions^59^. To rule out an effect of the Nnt mutation on the observed differences in VO_2_max between the BXD strains with the long and short *Cox7a2l* allele, we checked how the *Nnt* allele distributes between the two groups. We found an about equal distribution of DD (wt) and BB (null) *Nnt* allele among the two *Cox7a2l* genotypes (Extended Data Fig. 4c). Specifically, among the strains with the *Cox7a2l* DD allele, 45% have the *Nnt* BB allele and 50% have the *Nnt* DD allele. While among the strains with the *Cox7a2l* BB allele, 36% have the *Nnt* BB allele and 57% have the *Nnt* DD allele. Thus, the observed difference in VO_2_max is not due to differences in the *Nnt* gene.

**Fig. 5:**
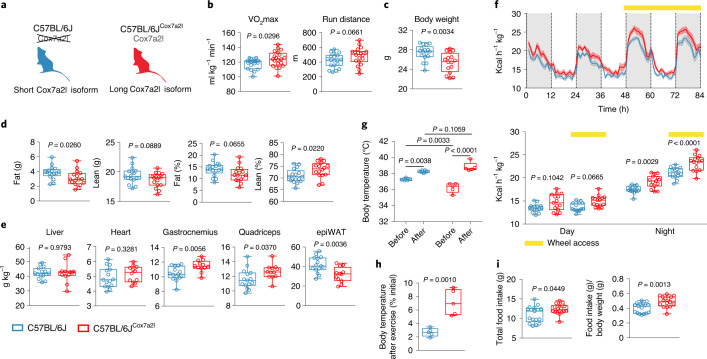
A *Cox7a2l* mutation affects cardiorespiratory fitness in mice. **a**, To dissect the specific role of *Cox7a2l*, we compared C57BL/6J mice expressing the long functional allele of *Cox7a2l* (red) with wild-type C57BL/6J mice (blue) expressing the non-functional short allele of *Cox7a2l* (ref. ^5^). **b**, VO_2_max and run distance (C57BL/6J *n* = 19, C57BL/6J^Cox7a2l^ *n* = 20). **c**, Body weight (C57BL/6J *n* = 17, C57BL/6J^Cox7a2l^ *n* = 16). **d**, Body composition measured by EchoMRI (C57BL/6J *n* = 17, C57BL/6J^Cox7a2l^ *n* = 16). **e**, Tissue weights normalized to total body weight (C57BL/6J *n* = 15, C57BL/6J^Cox7a2l^ *n* = 12). **f**, Energy expenditure measured using the Promethion metabolic cages in standard housing condition or with free access to a running wheel (yellow bar). Hour averages (top) and day and night averages (bottom) (C57BL/6J *n* = 15, C57BL/6J^Cox7a2l^ *n* = 15). **g**, Body temperature measured before and after 30 min of treadmill run (C57BL/6J *n* = 5, C57BL/6J^Cox7a2l^ *n* = 5). **h**, Body temperature shift after 30 min of treadmill run expressed as percentage of the initial body temperature (C57BL/6J *n* = 5, C57BL/6J^Cox7a2l^ *n* = 5). **i**, Food intake measured using Promethion metabolic cages. Total food intake (left) and food intake normalized to body weight (right) (C57BL/6J *n* = 15, C57BL/6J^Cox7a2l^ *n* = 15). Data are represented as mean ± s.e.m. (**f**, top) and box and whiskers (**b**–**i**). Box plot centers represent the median and lower and upper hinges represent the first and third quartiles (25th and 75th percentiles). The whiskers extend to the largest and smallest value. Statistical analysis was conducted by two-sided Student’s *t*-test (**b**–**e**,**h**,**i**), three-way ANOVA and Sidak’s multiple comparisons test (**f**) and two-way ANOVA and Sidak’s multiple comparisons test (**g**). Source data

**Extended Data Fig. 5:**
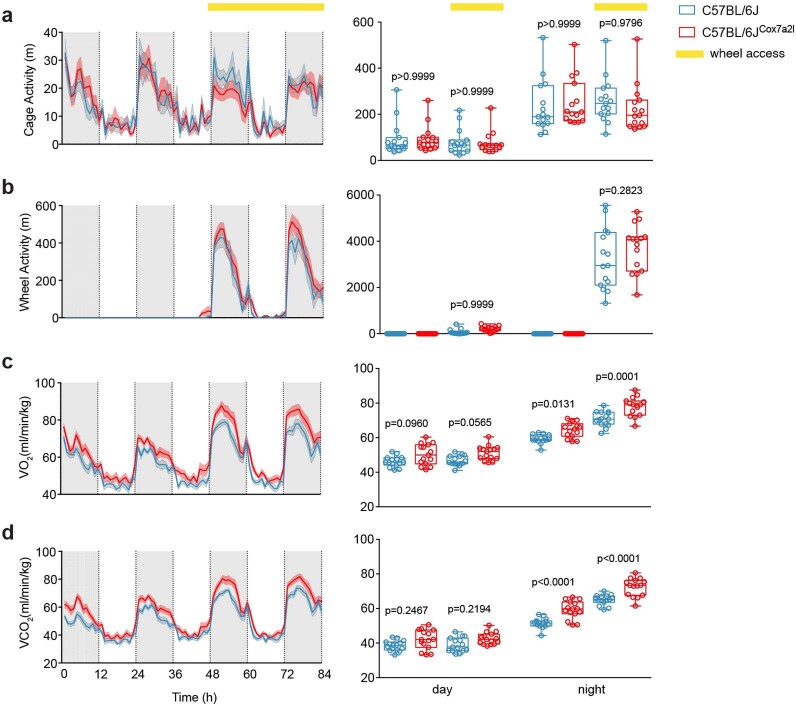
A spontaneous *Cox7a2l* mutation affects cardiorespiratory fitness in mice. Total cage activity (**a**), wheel running activity (**b**), VO_2_ (**c**) and VCO_2_ (**d**) measured using the Promethion metabolic cages in standard housing condition or with free access to a running wheel (yellow bar). **a-d**. Left panels: hour averages, right panels: day and night averages (C57BL/6 J n = 15, C57BL/6J^Cox7a2l^ n = 15). In **a-d** (left panels) data are represented as mean ± SEM. In **a-d** (right panels) data are represented as boxes and whiskers. Box plots center represents the median, lower and upper hinges represent the first and third quartile (25^th^ and 75^th^ percentiles). The whiskers extend to the largest and smallest value. Statistical analysis for **a-d**: three-way ANOVA and Sidak’s multiple comparisons test. Source data

To address the specific contribution of Cox7a2l to phenotypic differences, we used congenic C57BL/6J mice expressing the long (functional) form of *Cox7a2l* (thereafter referred to as C57BL/6J^Cox7a2l^)^5^ (Fig. 5a). Consistent with our previous findings in the BXD strains^26^ (Extended Data Fig. 4b), as well as with the phenotypic associations found with *COX7A2L cis*-eQTLs in the human genetic analysis of the UKBB, HBCS and HUNT datasets (Fig. 4b–e), C57BL/6J^Cox7a2l^ mice have higher VO_2_max (with a tendency for an increased run distance), lower body weight, more muscle mass and less fat deposits compared to C57BL/6J mice (Fig. 5b–e). To address Cox7a2l-driven metabolic differences, we housed C57BL/6J and C57BL/6J^Cox7a2l^ mice in Promethion metabolic cages with or without access to a running wheel (Methods). We did not observe changes in total activity and wheel activity between the two genotypes (Extended Data Fig. 5a,b). Notably, C57BL/6J^Cox7a2l^ mice had increased energy expenditure specifically during the active dark phase and this difference was more pronounced when mice had access to a running wheel (Fig. 5f and Extended Data Fig. 5c,d). While the body temperature of C57BL/6J^Cox7a2l^ was lower than C57BL/6J at rest, after exercise C57BL/6J^Cox7a2l^ had a higher body temperature increase (+2.6 °C versus +1 °C, respectively; Fig. 5g). This temperature shift was significantly higher also when expressing it as percentage of the initial body temperature (+7.1% versus +2.7%; Fig. 5h). These results suggest that mice with the long *Cox7a2l* allele can increase energy expenditure more when energy demands rise, compared to mice with the short *Cox7a2l* allele. Consistent with the observed increase in energy expenditure during the active phase, C57BL/6J^Cox7a2l^ mice had higher total and relative food intake (Fig. 5i), despite having lower body weight (Fig. 5c). Therefore, all together, these results confirm a specific role of *COX7A2L*/*Cox7a2l* in cardiorespiratory fitness in both human and mice.

### Cox7a2l-containing SCs are induced upon exercise

**Fig. 6:**
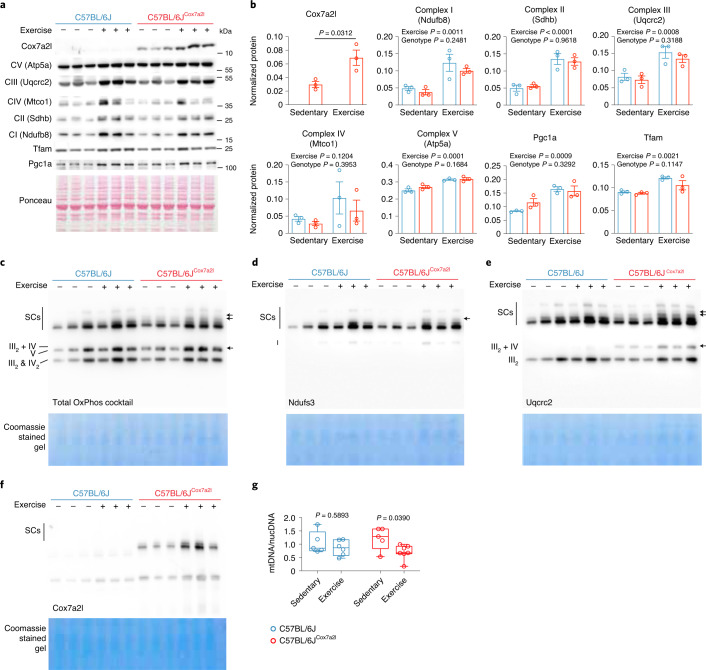
Cox7a2l-containing SCs are induced upon exercise. **a**, Western blot analysis of the indicated proteins at rest or after 5 weeks of wheel running exercise in gastrocnemius muscle. **b**, Western blot quantification of the results shown in **a**. **c**–**f**, BN–PAGE immunoblot with total OxPhos immunostaining (**c**), Ndufs3 immunostaining (**d**), Uqcrc2 immunostaining (**e**) and Cox7a2l immunostaining (**f**) in isolated mitochondria from gastrocnemius muscle at rest or after 5 weeks of wheel running exercise. Arrows represent the SCs recovered with the functional long allele of *Cox7a2l*. **g**, Mitochondrial-to nuclear DNA ratio measured in the gastrocnemius muscle at rest or after 5 weeks of wheel running exercise. Data are represented as mean ± s.e.m. (**b**) and box and whiskers (**g**). Box plot centers represent the median, lower and upper hinges represent the first and third quartiles (25th and 75th percentiles). The whiskers extend to the largest and smallest value. Statistical analysis was conducted by two-way ANOVA and Sidak’s multiple comparison. C57BL/6J sedentary *n* = 5, C57BL/6J exercise *n* = 6, C57BL/6J^Cox7a2l^ sedentary *n* = 5, C57BL/6J^Cox7a2l^ exercise *n* = 7. Source data

**Extended Data Fig. 6:**
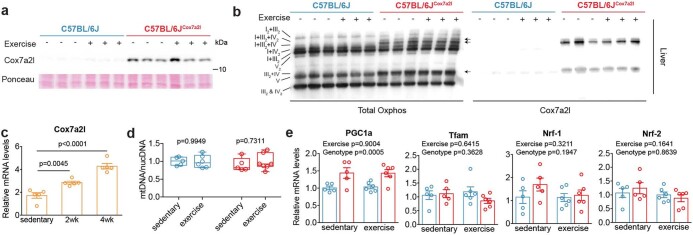
Cox7a2l is induced upon exercise specifically in the skeletal muscle. **a**. Western blot analysis of Cox7a2l protein expression at rest or after 5 weeks of wheel running exercise in liver. **b**. BN-PAGE immunoblot with total OxPhos immunostaining (left) and Cox7a2l immunostaining (right) in isolated mitochondria from liver at rest or after 5 weeks of wheel running exercise. **c**. Cox7a2l relative mRNA expression in the quadriceps of DBA/2 J mice in basal conditions (sedentary) or after 2 and 4 weeks of exercise training (n = 5). **d**. Mitochondrial to nuclear DNA ratio measured in liver at rest or after 5 weeks of wheel running exercise. **e**. mRNA expression of the indicated genes measured by qRT–PCR in gastrocnemius muscle at rest or after 5 weeks of wheel running exercise. In **c** and **e** data are represented as mean ± SEM. In **d** data are represented as box and whiskers. Box plots center represents the median, lower and upper hinges represent the first and third quartile (25^th^ and 75^th^ percentiles). The whiskers extend to the largest and smallest value. Statistical analysis for **c**: one-way ANOVA and Dunnett’s multiple comparisons test, statistical analysis for **d** and **e**: two-way ANOVA and Sidak’s multiple comparison test. Source data

Given the specific role of COX7A2L in cardiorespiratory fitness and energy expenditure during physical activity and as SCs are induced by exercise in humans^24^, we addressed the role of *Cox7a2l* in exercise. C57BL/6J and C57BL/6J^Cox7a2l^ mice were given free access to a running wheel for 5 weeks and then killed. Both Cox7a2l total protein and Cox7a2l-containing SCs were increased in the skeletal muscle of C57BL/6J^Cox7a2l^ mice after 5 weeks of training, but were absent in C57BL/6J mice (Fig. 6a–f). Notably, only C57BL/6J^Cox7a2l^ mice showed an induction of the higher respirasome bands and III_2_ + IV SC upon exercise (Fig. 6c–e). This increase was likely muscle tissue-specific as no changes were observed in Cox7a2l protein expression and the levels of Cox7a2l-containing SCs upon exercise in the liver (Extended Data Fig. 6a,b). The increase in Cox7a2l expression upon exercise was not dependent on the genetic background, as it was also observed in DBA/2J mice and was proportional to the duration of the training (Extended Data Fig. 6c). Given the observed association of Cox7a2l with other mitochondrial ontologies (Fig. 1a) we assessed possible differences in mitochondrial gene expression and content in the two genotypes. We measured levels of individual mitochondrial complexes by western blot and measured PGC1a and TFAM as markers of mitochondrial biogenesis both at mRNA and protein levels (Fig. 6a,b and Extended Data Fig. 6e). We found an overall increase in the amount of mitochondrial complexes as well as PGC1a and TFAM protein levels upon endurance exercise, this was expected as endurance exercise is known to increase mitochondrial biogenesis (Fig. 6a,b). The increase in the mitochondrial complexes, PGC1a and TFAM was equal in both genotypes. Thus, upon exercise, the observed increase in the Cox7a2l-containing respirasome bands in the mice expressing the long isoform of Cox7a2l is not simply due to an overall increase in the single complexes but depends on Cox7a2l. Furthermore, C57BL/6J^Cox7a2l^ mice, but not C57BL/6J mice, had decreased mitochondrial DNA (mtDNA)/nuclear DNA (nDNA) ratio in the muscle (but not the liver) after exercise (Fig. 6g and Extended Data Fig. 6d). This phenomenon was also observed in humans^60,61^ and might suggest increased mitochondrial turnover and remodeling with long-term training (Discussion). Altogether, these data suggest a Cox7a2l-dependent increase in respirasome formation upon exercise.

## Discussion

The assembly of MRCs in SCs is widely accepted; however, their functional importance has so far remained elusive. While several studies have found that SC assembly may be beneficial to enhance electron transport efficiency, channel substrates, reduce ROS production and stabilize single MRCs^4,8,9,11–13,15,62^, other studies have challenged these observations^63,64^. COX7A2L is required for the interaction of complex III with complex IV and is important for the formation of the respirasome^5,26^; however, these findings have also been contested^31,32,65–68^. Most structural models of the mammalian respirasome come from mitochondria isolated from heart at rest; however, it is now clear that SC assembly and the role of assembly factors may be dependent on the tissue type and metabolic status of the cell. Although only a few studies have investigated the physiological function of COX7A2L, a recent study suggested that it may promote metabolic efficiency in zebrafish and mice^25^. Nonetheless, the possible function COX7A2L in cellular bioenergetics is still controversial^30^ and its role in human is unknown.

To shed light on this conundrum, we sought to determine the function of COX7A2L in human. We found that genetic variation strongly affects COX7A2L expression. We identified the highest number and most significant *cis*-eQTLs in the skeletal muscle. Although several other tissues also had a high number of significant *cis*-eQTLs with high overlap between tissues, the effect of the alternative allele was opposite in muscle tissues compared to most other tissues. This suggests a muscle-specific mechanism mediating the effect of the genetic variant on gene expression. COX7A2L lead *cis*-eQTL is a 3′ UTR insertion that is sufficient by itself to increase expression in cultured cells. We identify a possible mechanism by which this 3′ UTR variant affects expression. The insertion creates a short-repeated sequence that increases mRNA stability, possibly through increased binding of an mRNA stabilizing factor or through disruption of a microRNA binding site. Further investigations are needed to identify the detailed molecular mechanism and to determine why this variant might have a negative effect on gene expression in most other tissues such as the liver and thyroid. In our analysis we found other independent variants that affect COX7A2L expression, thus we cannot exclude that other independent causal variants exist and act through different mechanisms.

Increased *COX7A2L* expression in human myoblasts leads to increased amounts of SCs and increased cellular respiration when cells are cultured in galactose medium; this is in line with previous reports showing that SCs may play a role in certain metabolic stress conditions and that COX7A2L may be important when cells are forced to use oxidative phosphorylation to produce ATP^27^. Physical exercise causes metabolic stress in muscle tissues. The increased energy demand from muscle contraction leads to a surge in cellular AMP levels and to a decline in oxygen levels that triggers mitochondrial adaptations to decreased energy levels and hypoxia. We show that Cox7a2l is induced in skeletal muscle upon exercise in mice and that humans with higher expression of COX7A2L have improved metabolic and cardiorespiratory phenotypes. While some studies have demonstrated a rearrangement of mitochondrial RCs into supercomplexes upon exercise training^24,69^, one recent study reported that increased mitochondrial biogenesis, rather than rearrangement of mitochondrial complexes into supercomplexes is responsible for improved muscle bioenergetics upon exercise^70^. Here, while we observe an increase in mitochondrial biogenesis and in the amount of OxPhos complexes upon exercise in mice, we demonstrate that the absence of Cox7a2l protein and the lack of respirasome formation affect exercise performance and energy expenditure. Of note, mice expressing Cox7a2l have a decreased mtDNA/nDNA ratio after exercise. mtDNA synthesis is an energy-consuming process that may be less favored in situations of reduced cellular ATP, such as during or after physical effort. Furthermore, exercise leads to increased ROS production and oxidative stress in the muscle that may damage mtDNA^71,72^. Thus, it is plausible that improved mitochondrial efficiency, associated with mitochondrial turnover and remodeling, rather than overall increase in mtDNA synthesis, may be favored in certain conditions of metabolic stress. Such a decrease in mtDNA/nDNA ratio in skeletal muscle upon exercise was also observed in humans^60,61^, suggesting a conserved adaptive response. Our findings suggest that individuals having genetic variants associated with increased COX7A2L expression in the muscle may be able to better adapt to situations of increased metabolic stress and physical effort demand, such as during physical activity. Notably, we found that the frequency of *COX7A2L* lead eQTL is remarkably different in certain populations (Extended Data Fig. 2a), suggesting a possible adaptive role of SCs in different environmental conditions; however, further research is needed to support this hypothesis. Previous studies have identified several human genetic polymorphisms in nuclear and mtDNA affecting mitochondrial gene expression^73^, metabolism^74^ and exercise performance^75,76^ as well as exercise-induced changes in mitochondrial gene expression^77^. Co-inheritance of these genetic polymorphisms with the *COX7A2L* variants may be used in the future for metabolic risk prediction.

The findings in our study not only shed light on the genetic underpinnings of how variants in *COX7A2L* affect mitochondrial supercomplex formation in human muscle, but also demonstrate the importance of COX7A2L and SCs for cardiometabolic fitness and human health. Cardiorespiratory fitness is an important predictor of cardiovascular and metabolic health^78,79^. Improving cardiorespiratory fitness and exercise capacity is a highly promising approach to reduce all-cause and cardiovascular mortality^79^.

## Methods

### Helsinki Birth Cohort

The HBCS includes 13,345 individuals born in Helsinki between 1934 and 1944. The clinical study includes 2003 participants and the protocol was approved by the Ethics Committee of Epidemiology and Public Health of the Hospital District of Helsinki and Uusimaa. Written informed consent was obtained from each participant before any study procedure was initiated. The UKK 2-km walk test has been validated against maximal effort tests by treadmill or bicycle ergometry in multiple populations, including individuals with obesity^53^ and elderly individuals^54^. During the test, participants are instructed to walk a 2-km course on flat ground as fast as possible. The test results are expressed as a fitness index, which takes into account the individual’s age, sex, BMI, time spent in walking and heart rate. The senior fitness test, which describes the physical fitness of the study participants was performed in 695 individuals and was described previously^80^. Isometric grip strength of the dominating hand was tested by a Newtest Grip Force dynamometer (Newtest Oy). The maximum value of three squeezes was used in analyses of linear regression. Weight and height were measured at the time of the grip strength measurements and BMI was calculated as weight (kg) divided by height (m) squared.

DNA was isolated from blood and genotyping was carried out with the modified Illumina 610k chip by the Wellcome Trust Sanger Institute. Genotype imputation was performed using the 1000 Genomes Project Phase I integrated variant set (v.3/April 2012; NCBI build 37/hg19) and IMPUTE2 software. Before genotype imputation some quality control filters were applied: SNP clustering probability for each genotype >95%; call rate >95% individuals and markers (99% for markers with MAF < 5%); MAF > 1%; and Hardy–Weinberg equilibrium *P* > 1 × 10^−6^. Moreover, heterozygosity, sex checks and relatedness checks were performed and any discrepancies were removed. Phenotype association analyses in HBCS were performed in individuals who had available genotype and senior fitness test data, approximately 400 individuals for the analysis. The scores of the fitness tests were classified based on fifth percentile range, with a score of 1 being the worst performance (score below fifth percentile); 2 the score from the fifth to ninth percentile; and 20 the best performance (in or above the 95th percentile) as described^50^. We performed linear regressions with SNPtest^81^ assuming an additive genetic model. We adjusted all models for age, sex, highest education achieved (basic or less/upper secondary/lower tertiary/upper tertiary) and smoking (yes/no).

### The Trøndelag Health study

The HUNT study and HUNT3 sub-study (HUNT3 fitness study^47,48^) were described previously^48^. The HUNT3 fitness study was approved by the Regional Committee for Medical Research Ethics (2012/1672/REK nord), the Norwegian Data Inspectorate and the National Directorate of Health and is in compliance with the Helsinki Declaration. Written informed consent was obtained from all participants. The 4,631 healthy adult participants performed a measure of VO_2_max. Exclusion criteria for the HUNT3 fitness study were described previously^48^. VO_2_max measurement for this study was described previously^48^. The 4,463 participants who reached VO_2_max were included in the analyses.

DNA was extracted from blood samples, as described previously^82^. Genotyping of the HUNT study was described previously^83^. In brief, genotyping was performed with one of three different Illumina HumanCoreExome arrays (HumanCoreExome12 v.1.0, HumanCoreExome12 v.1.1 and UM HUNT Biobank v.1.0) according to standard protocols. The genotypes from different arrays had quality control performed separately and were reduced to a common set of variants across all arrays. Samples that failed to reach a 99% call rate, had contamination >2.5% as estimated by BAF Regress, large chromosomal copy number variants, lower call rate of a technical duplicate pair and twins, gonosomal constellations other than XX and XY or whose inferred sex contradicted the reported sex were excluded. The genomic position, strand orientation and reference allele of genotyped variants were established by aligning the probe sequences against the human genome using the BLAT tool^84^. With PLINK v.1.90 (ref. ^85^), variants were excluded if probe sequences could not be perfectly mapped and cluster separation was <0.3, Gentrain score <0.15, showed divergence from Hardy–Weinberg equilibrium in unrelated samples of European ancestry (*P* < 0.0001), had call rates <99% or another array with higher call rate genotyped the same variant. Ancestry of samples was derived from projecting genotyped samples into the principal components of the Human Genome Diversity Project reference panel. Different arrays were matched by narrowing to a set of overlapping variants and eliminating variants with frequency differences >15% between datasets, or that were monomorphic in one and had MAF > 1% in another dataset. These genotype data were phased using Eagle2 (v.2.3.8)^86^. Imputation was performed on samples of recent European ancestry using Minimac3 (v.2.0.1; http://genome.sph.umich.edu/wiki/Minimac3)^87^ and a merged reference panel built on the Haplotype Reference Consortium panel^88^ (release v.1.1) together with a local reference panel, which included 2,202 whole-genome sequenced HUNT study participants. Relatively unrelated individuals (kinship coefficient <0.0884) were chosen using KING^89^ and FastIndep^90^ tools. BOLT-LMM algorithm^91^ was employed to execute statistical analyses while variants with MAF < 0.0011^92^ were excluded from the analysis. This algorithm computes statistics for testing association between phenotype and genotypes using a linear mixed model. The analysis was adjusted for sex and age. Genetic principal components were included as covariates in the analysis to control for residual population structure. The study was in conformity with Norwegian laws and the Helsinki Declaration and signed informed consent was obtained from all participants.

### Mouse lines

C57BL/6J mice expressing the long functional *Cox7a2l* allele were generated as described^5^. The C57BL/6J OlaHsd used to generate the C57BL/6J^Cox7a2l^ mice carried the wild-type allele of the Nnt gene. For genotyping, DNA was extracted from tails and PCR amplified using the KAPA2G mix (KK5103, Kapa Biosystems) following manufacturer’s instructions (Supplementary Table 6 lists the primer sequences). PCR products were run on a QIAxcel instrument to identify different molecular weight bands indicating presence or absence of the six-nucleotide deletion (50 or 56 bp). For all experiments, littermates of the same sex were randomly assigned to experimental groups. All experiments were performed on male mice at 10–14 weeks of age. All mice were fed a chow diet (SAFE 150) and were kept in standard housing conditions (12-h light–12-h dark cycle and temperature of 22 °C). All animals had ad libitum access to food and water. All animal experiments were performed according to Swiss ethical guidelines and were approved by the Service de la Consommation et des Affaires Vétérinaires of the Canton de Vaud (license VD3419)

### Cell lines

All cell lines were cultured in a humidified incubator at 37 °C and 5% CO_2_. HEK-293T (CRL-3216, ATCC) cells were cultured in Dulbecco’s modified Eagle’s medium (DMEM; Gibco, 41966029) supplemented with 10% fetal bovine serum (Gibco, 10270106) and 1% penicillin/streptomycin (Gibco, 15140122). Human myoblast lines from non-diseased individuals were obtained from the CBC BioTec Centre de Biologie et Pathologie Est, Groupement Hospitalier Est and were maintained at a maximum confluency of 70% in myoblast culture medium (Ham’s F-10 Nutrient Mix (Gibco, 31550031), 12% fetal bovine serum and 1% penicillin/streptomycin). For differentiation, myoblasts were seeded in culture medium and after 3 d, when they reached ~80–90% confluence, the culture medium was replaced with differentiation medium (DMEM/F12 (Gibco, 31330038), 2% horse serum (Gibco 16050122) and 1% penicillin/streptomycin (Gibco, 15140122)). Trypsin-EDTA 0.05% (Gibco, 25300062) was used for detachment.

### DNA extraction and genotyping of cell lines

DNA extraction was performed using NucleoSpin Tissue mini kit (Macherey-Nagel, 740952). For genotyping, primers flanking the rs4181 10-bp insertion site were used for PCR amplification (primer sequences are listed in Supplementary Table 6). PCR was performed using the KAPA2G mix (KK5103, Kapa Biosystems) following manufacturer’s instructions. PCR products were run on a QIAxcel instrument to identify different molecular weight bands indicating presence or absence of the insertion (425 or 435 bp). PCR products were further purified using the PCR clean-up gel extraction kit (Macherey-Nagel, 740609) and sequenced by Sanger sequencing (Microsynth) to confirm electrophoresis results. Only homozygous myoblast lines were used for experiments.

### GeneBridge analysis

Ontology terms and pathways correlated to *COX7A2L* in humans were identified using the publicly available GeneBridge tool (https://systems-genetics.org). First, an analysis was performed with all available tissues in the database. GO terms and pathways with GMAS ± 0.268 were considered significantly associated with *COX7A2L*; the threshold of significance was determined as previously described^33^ (Fig. 1a). Then, 13 main tissues were selected and the same analysis was repeated for each tissue separately. GO terms and pathways with score ± 0.268 in at least one of the 13 tissues are represented in Extended Data Fig. 1a.

### Genotype-Tissue Expression analyses

For the *cis*-eQTL analysis we used publicly available data from the GTEx Consortium^34^ (https://www.gtexportal.org) v.8 release. Only *cis*-eQTLs (eQTLs within 1 Mb from the *COX7A2L* gene) are included in the analysis. For the correlation analyses in GTEx, we obtained the publicly available gene transcripts per million and the covariates used in the *cis*-eQTL analysis (dbGAP study accession no. phs000424.v8). To avoid correlations due to known or unknown factors and batch effects, we used the removeBatchEffect effect function from the limma R package v.3.38.3 (ref. ^93^) along with covariates as calculated and released by the GTEx consortium.

### Mendelian randomization

For the Mendelian randomization analysis, we made use of the *cis*-eQTL data from GTEx^34^, which are freely available from https://storage.googleapis.com/gtex_analysis_v8/single_tissue_qtl_data/GTEx_Analysis_v8_eQTL.tar. rs4181 was not genotyped in the UKBB and the HBCS, therefore, we selected the second-most-significant muscle *cis*-eQTL, rs10183278, as the instrumental variable for our analysis. This *cis*-eQTL is in high LD with rs4181 (*r*^2^ = 0.952). As with rs4181, rs10183278 is also significantly linked with *EML4* expression, a gene whose 3′ terminus lies only 997 bp upstream of *COX7A2L*, but this association is much weaker; the effect size on *COX7A2L* expression in muscle is 0.44 (*P* = 3.7 × 10^−68^) versus an effect size of −0.12 for *EML4* (*P* = 2.6 × 10^−5^). We also verified rs10183278 in PhenoScanner v.2 (http://www.phenoscanner.medschl.cam.ac.uk/)^94^ and found no obvious confounders. The results of this analysis are in Supplementary Table 7. Muscle gene expression of *COX7A2L* was used as the exposure variable. We used the available_outcomes function from the TwoSampleMR v.0.5.3 R package to obtain 188 body weight- and health-related outcome summary statistics from the IEU GWAS database (https://gwas.mrcieu.ac.uk/datasets/)^41^ (Supplementary Table 5). For the HBCS, we used the summary statistics of the additive genetic model (see section on Helsinki Birth Cohort). Statistical inference was performed through Wald ratio tests as implemented in the harmonise_data function of the TwoSampleMR package and *P* values were converted to *q* values with the Benjamini–Hochberg FDR procedure.

### Body composition

Whole-body composition was measured by NMR using a Minispec instrument (Bruker).

### Metabolic cages

Energy expenditure, VO_2_, VCO_2_, food intake, cage and wheel activity were measured using the Promethion system (Sable Systems International). Mice were housed in metabolic cages for 4 d and had free access to a running wheel. To measure energy expenditure and cage activity in standard housing conditions, the wheel was kept blocked for the first 2 d of recording.

### VO_2_max test in mice

VO_2_max in mice was measured with a calorimetric treadmill (Columbus Instruments) with an incremental speed protocol. To avoid anxiety created by finding themselves in a new environment, mice were habituated to the device for 10 min before starting the test. The run distance, VO_2_ and VCO_2_ were measured. The experiment was stopped when mice were exhausted or VO_2_max was reached (when VO_2_ levels failed to increase despite increasing running velocity or when respiratory exchange ratio was =1.0).

### Long-term training

For long-term training experiments mice were housed singularly and had access to a running wheel for 5 weeks. After a first estimation of the average distance run per day by the first mouse cohort, the maximum allowed distance was set on this average (1,800 m per day) after which the wheel was blocked for all mice to avoid large variability between mice and between experiments. The meters run on the wheel was monitored for each mouse and for all the duration of the experiment. There were no significant differences in the total distance run between the two genotypes. Mice in control groups were also housed singularly and had access to a wheel; however, the wheel was blocked for the entire duration of the experiment.

### Treadmill exercise and body temperature measurement

Body temperature was measured using a rectal probe before and after 30 min of treadmill run at incremental speed (10 min at 20 cm s^−1^, 10 min at 23 cm s^−1^ and 10 min at 26 cm s^−1^). The mice were acclimatized to the treadmill for 3 d before the experiment. All animals satisfactorily reached the end of the test.

### BN–PAGE immunostaining

BN–PAGE was performed as described^95,96^ with some modifications. Briefly, ~15–30 mg of frozen tissue or ~20 × 10^6^ cells were homogenized to isolate mitochondria and mitochondria protein content was quantified as described^96^. For BN–PAGE immunoblotting, 50 μg (liver and muscle) or 10 μg (myotubes) of mitochondria extract was loaded in the gel. Electrophoresis of solubilized mitochondrial proteins was performed as described^96^. For immunoblotting, samples were run at 150 V for 30 min and at 250 V for additional 90 min. After electrophoresis the gel was soaked for 30 min in Tris/glycine transfer buffer (25 mM Tris, 192 mM glycine, 10% methanol and 0.1% SDS). Proteins were transferred in Tris/glycine transfer buffer on a PVDF membrane using a wet transfer system (Bio-Rad). Blocking and antibody incubations were performed in 3% BSA diluted in TBS-T. Membranes were incubated with primary antibodies: total OXPHOS Rodent WB Antibody Cocktail (Abcam, ab110413, 1:1,000 dilution), anti-NDUFS3 (Abcam, ab14711, 1:2,000 dilution), anti-UQCRC2 (Abcam, ab14745, 1:10,000 dilution), anti-MTCO1 antibody (Abcam, ab14705, 1:1,000 dilution) and anti-COX7A2L antibody (St John’s Laboratory, STJ110597, 1:1,000 dilution) to detect Cox7a2l-containing SC bands. Anti-SDHA antibody (Abcam, ab14715, 1:1,000 dilution) was used as loading control. Protein signal was detected using chemiluminescence (WesternBright ECL, Advansta) and imaged using the Fusion FX6 imaging system (Vilber).

### RNA extraction and qRT–PCR

RNA was extracted using TriPure Isolation Reagent (Roche, 11667165001). For the RNA extraction from myoblasts and differentiated myotubes, two wells of a six-well plate were pooled for each sample and 1 μl of glycogen (R0551, Thermo Fisher) was added during the extraction for maximum recovery of the RNA and visualization of the pellet. For reverse transcription, PrimeScript RT Reagent kit with gDNA Eraser (Takara, RR047B) was used following manufacturer’s instructions. For qPCR TB Green Premix Ex Taq (Takara, RR420W) was used and plates were run on a LightCycler 480 instrument (Roche). Primer sequences are listed in Supplementary Table 6. All qRT–PCR fold changes were calculated using *Gapdh*/*GAPDH* as housekeeping gene. The average of three technical replicates was used for each data point.

### MtDNA/nucDNA ratio

MtDNA abundance was measured with the ΔΔCt method as previously described^97^. Briefly, ~15 mg of frozen liver or muscle tissues were used to extract total cellular DNA using NucleoSpin Tissue kit (Macherey-Nagel, 740952). For qPCR, 20 ng of DNA was used. qPCR was carried out as described above. For mtDNA amplification, primers against the 16S rRNA were used (Supplementary Table 6). For nuclear DNA amplification primers against the β2M gene were used (Supplementary Table 6). For mtDNA copy number estimation in human myoblasts the Human Mitochondrial DNA Monitoring Primer Set (Takara, 7246) was used.

### Western blot

For tissue samples, ~10 mg of frozen tissue were homogenized in RIPA buffer with protease inhibitors (Thermo Fisher, 78429) using a small pestle, mixed for 20 min at 4 °C and then spun for 20 min at 11,481 r.p.m. at 4 °C to eliminate cell debris. For myotube samples, three wells of a six-well plate were pooled together for one replicate and resuspended in RIPA buffer with protease inhibitors. Samples were incubated on ice, sonicated briefly to ensure complete cell lysis and spin for 20 min at 11,481 r.p.m. at 4 °C to eliminate cell debris. Protein amount was quantified using the DC protein assay (Bio-Rad). Proteins were separated by SDS-polyacrylamide gel electrophoresis and transferred onto PVDF membranes using a wet transfer system (Bio-Rad). Blocking and antibody incubations were performed in 5% BSA diluted in TBS-T. The following primary antibodies were used: anti-Cox7a2l (St John’s Laboratory, STJ110597 and Proteintech, 11416-1-AP, 1:1,000 dilution), total OXPHOS Rodent WB Antibody Cocktail (Abcam, ab110413, 1:1,000 dilution), anti-Vinculin (Abcam, ab129002, 1:1,000 dilution), anti-TFAM (Abcam, ab131607, 1:1,000 dilution), anti-PGC1a (Calbiochem, ST1202, 1:1,000 dilution) and anti-VDAC1 (Abcam, ab14734, 1:1,000 dilution). Protein signal was detected using chemiluminescence (WesternBright ECL, Advansta) and imaged using the Fusion FX6 imaging system (Vilber).

### Luciferase reporter assay

A 100-bp fragment of the human *COX7A2L* 3′ UTR surrounding the rs4181 insertion variant with (3′ UTR-ALT) or without (3′ UTR-REF) the 10-bp insertion was cloned in a pUC57 vector by Genscript (for the insert sequence see Supplementary Table 6) flanked by XbaI and FseI restriction sites at the 5′ and 3′ respectively. The 100-bp fragment was excised and cloned in the pGL3-promoter vector (Promega) downstream of the luciferase gene using XbaI and FseI restriction enzymes (NEB). The obtained plasmids were sequenced to check the correct directionality of the inserted sequence (Microsynth). For the luciferase assay, HEK-293T cells were transfected in 96-well plates with pGL3-promoter-3′ UTR-REF, pGL3-promoter-3′ UTR-ALT or pGL3-promoter-empty vectors (190 ng per well) together with a pRL-CMV vector (10 ng per well) to control for transfection efficiency. Transfection was performed using JetPEI transfection reagent (Polyplus) with the reverse transfection method following manufacturer’s instructions. Briefly, cells were trypsinized, the JetPEI-DNA mix was added to the cell suspension and ~30,000 cells were then plated per well. A JetPEI-DNA ratio of 2:1 was used. At 24 h after transfection both luciferase and Renilla luminescence was measured using the Dual-Glo Luciferase Assay System (Promega, E2920). A minimum of eight wells per conditions were measured.

### Seahorse cellular respiration analysis

For cellular respiration analysis myoblasts lines were seeded at 80–90% confluence in growth medium and the following day they were switched to differentiation medium (see above). Myoblasts were differentiated for at least 7 d. At 48 h before the experiment the medium was switched to glucose-free medium (DMEM, no glucose (Gibco, 11966025) supplemented with 10 mM galactose (Sigma, G0750), 1 mM sodium pyruvate (Gibco, 11360070), 2% horse serum (Gibco 16050122) and 1% penicillin/streptomycin (Gibco, 15140122)) or high-glucose medium (DMEM, high-glucose (Gibco, 11965092) supplemented with 1 mM sodium pyruvate (Gibco, 11360070), 2% horse serum (Gibco 16050122) and 1% penicillin/streptomycin (Gibco, 15140122)). OCR was measured using a Seahorse analyzer (XF96, Agilent Technologies), following the manufacturer’s instructions. For the glucose-free condition, XF base medium (102353-100, Agilent Technologies) was supplemented with 10 mM galactose (Sigma, G0750), 1 mM sodium pyruvate (Gibco, 11360070) and 4 mM GlutaMAX (Gibco, 35050061). For the high-glucose condition, XF base medium (102353-100, Agilent Technologies) was supplemented with 25 mM glucose (Sigma, G8270), 1 mM sodium pyruvate (Gibco, 11360070) and 4 mM GlutaMAX (Gibco, 35050061). After measuring basal respiration, 1 μM oligomycin was added to inhibit complex V (ATP-synthase) and measure ATP-linked respiration. Maximal respiration was measured by adding 2 μM FCCP, a strong uncoupling agent. Finally, 1 μM rotenone/antimycin A mix was added to inhibit mitochondrial respiration.

### COX7A2L promoter tagging with dCAS9-HA

COX7A2L promoter tagging was performed as described earlier^98^ with some modifications. Guide RNAs for *COX7A2L* promoter were designed using the online GPP web portal tool (https://portals.broadinstitute.org/gpp/public/analysis-tools/sgrna-design-crisprai?mechanism=CRISPRa) using *Streptococcus* *pyogenes* PAM sequence (NGG). Two gRNAs having the best predicted on- and off-target scores were selected (gRNA1and gRNA2; see also Supplementary Table 6). Both gRNAs were cloned into phU6 plasmids using BbsI restriction enzyme (Genewiz). Insertion of the gRNA was verified by sequencing (Microsynth). HEK-293T cells cultured in 15-cm dishes were co-transfected with 75 μg of dCas9-HA (Addgene, 61355) and 27 μg of the empty phU6 plasmid (EV) or its subclone expressing the gRNA sequence. Transfection was performed using Lipofectamine 3000 (Invitrogen, L3000001). At 24 h after transfection, Opti-MEM medium (Gibco, 31985070) was replaced with normal culture medium and 24 h later cells were collected for chromatin immunoprecipitation. gRNA1 gave the highest enrichment for *COX7A2L* promoter and was therefore selected for the experiment in Extended Data Fig. 2c.

### Chromatin immunoprecipitation followed by qPCR

Chromatin immunoprecipitation was performed as described^39^ with some modifications. Cells expressing dCas9-HA and gRNA or EV were washed once with 1× PBS and crosslinked with 1% PFA for 10 min at room temperature. Crosslinking was quenched by adding glycine at a final concentration of 0.125 M. Cells were rinsed twice with 1× PBS and collected. After centrifugation at 1,200 r.p.m. for 6 min at 4 °C, the cell pellet was resuspended in lysis buffer 1 (50 mM HEPES-KOH, pH 7.5, 140 mM NaCl, 1 mM EDTA, pH 8.0, 10% glycerol, 0.5% NP-40, 0.25% Triton X-100 and protease inhibitors (Thermo Fisher Scientific, 78430)) and rotated for 10 min at 4 °C. After centrifugation at 1,200 r.p.m. for 6 min at 4 °C, the cell pellet was resuspended in lysis buffer 2 (10 mM Tris-HCl, pH 8.0, 200 mM NaCl, 1 mM EDTA, pH 8.0, 0.5 mM EGTA, pH 8.0 and protease inhibitors). Following centrifugation at 1,200 r.p.m. for 6 min at 4 °C, the cell pellet was resuspended in lysis buffer 3 (10 mM Tris-HCl, pH 8.0, 100 mM NaCl, 1 mM EDTA, pH 8.0, 0.5 mM EGTA, pH 8.0, 0.1% Na-deoxycholate, 0.5% *N*-lauroylsarcosine and protease inhibitors). Chromatin was sheared by sonication with diagenode bioruptor UCD-200 (three 5-min cycles of 30 s ON + 30 s OFF on middle strength). Chromatin was centrifuged at 20,000*g* for 30 min at 4 °C to remove cell debris. Two percent of the supernatant was stored at −80 °C to be used as input. Each sample was divided in two and incubated with 5 μg of either anti-hemagglutinin (ab9110) or IgG control antibody (sc2025). Samples were rotated at 4 °C overnight. For immunoprecipitation, Dynabeads Protein A Immunoprecipitation kit was used (Invitrogen, 10006D). Then, 50 μl of magnetic beads per sample were washed twice with 200 μl of antibody binding and washing buffer and twice with 200 μl of lysis buffer 3. Samples were added to the beads and rotated at 4 °C for 4 h. Beads were then washed three times with 150 mM wash buffer (1% Triton X-100, 0.1% SDS, 150 mM NaCl, 2 mM EDTA, pH 8.0 and 20 mM Tris-HCl, pH 8.0) and twice with 500 mM wash buffer (1% Triton X-100, 0.1% SDS, 500 mM NaCl, 2 mM EDTA, pH 8.0 and 20 mM Tris-HCl, pH 8.0). After the washes, the beads were resuspended in 120 μl of elution buffer (1% SDS and 0.1 M NaHCO_3_) and incubated for 1 h at 65 °C with shaking. The supernatant was collected in new tubes and both supernatant and input were incubated overnight at 65 °C to reverse crosslinking. Immunoprecipitated DNA was purified using MinElute PCR Purification kit (QIAGEN, 28004) and assayed by qPCR. For qPCR, LightCycler 480 SYBR Green I Master (Roche, 04887352001) and primers were added to 3 μl of the purified DNA. Primer sequences are listed in Supplementary Table 6. Fold enrichment over IgG was calculated based on the percent input recovered in anti-hemagglutinin and IgG samples.

### RNA stability assay

The RNA stability assay in differentiated myotubes was performed as described previously^99^. Briefly, myoblasts were grown in differentiation medium for 10 d, at day 10 of differentiation, actinomycin D (Sigma, A1410) was added to the medium at a concentration of 10 μg ml^−1^ and cells were collected in TriPure Isolation Reagent (Roche, 11667165001) after 0 and 1 h of treatment. RNA extraction and qRT–PCR were performed as described above. RNA abundance at each time point was calculated relatively to time 0.

### Quantification and statistical analysis

No statistical methods were used to predetermine sample size. The exact value of *n*, the statistical methods used to determine significance and error bars are described in the figure legends. All replicates represent biological replicates. A two-tailed Student’s *t*-test was used to calculate statistical differences between the means of two groups. One-way or two-way ANOVA tests were used to determine statistical differences between multiple groups. Statistical tests were performed using GraphPad Prism 7 or R. In the Promethion experiment, mice that did not spontaneously run on the running wheel were excluded from the analysis (*n* = 1–2 per group).

### Materials availability

Plasmids generated in this study will be made available upon request to the corresponding author.

### Reporting summary

Further information on research design is available in the Nature Research Reporting Summary linked to this article.

## Supplementary information

Reporting Summary

Supplementary Tables 1–7 Supplementary Table 1. COX7A2L correlated modules. Supplementary Table 2. All significant COX7A2L *cis*-eQTLs. Supplementary Table 3. Tissue abbreviations. Supplementary Table 4. rs4181 allele frequencies. Supplementary Table 5. Mendelian randomization analysis. Supplementary Table 6. Oligonucleotide sequences. Supplementary Table 7. PhenoScanner analysis.

## Data Availability

The data generated in this study are available with the published manuscript and/or were deposited at Mendeley Data (10.17632/8fcntj63x6.1) and will be publicly available upon publication. The GTEx gene expression and eQTL data are publicly available at http://www.gtexportal.org/home/. GO terms and pathways correlated to COX7A2L can be found through the publicly available GeneBridge tool (https://systems-genetics.org). Genotype frequencies data from the 1000 Genomes Project Phase 3 and gnomAD are publicly available at https://www.ensembl.org/. The IEU GWAS database is publicly available at https://gwas.mrcieu.ac.uk/datasets/. Source data are provided with this paper.

## Source data

Source Data Fig. 2 Statistical Source Data.
Source Data Fig. 3 Statistical Source Data.
Source Data Fig. 3 Unprocessed western blots and/or gels.
Source Data Fig. 5 Statistical Source Data.
Source Data Fig. 6 Statistical Source Data.
Source Data Fig. 6 Unprocessed western blots and/or gels.
Source Data Extended Data Fig. 2 Statistical Source Data.
Source Data Extended Data Fig. 3 Statistical Source Data
Source Data Extended Data Fig. 3 Unprocessed western blots and/or gels.
Source Data Extended Data Fig. 4 Statistical Source Data.
Source Data Extended Data Fig. 5 Statistical Source Data.
Source Data Extended Data Fig. 6 Statistical Source Data.
Source Data Extended Data Fig. 6 Unprocessed western blots and/or gels.

## Code availability

Essential scripts used in this study were deposited at Mendeley Data (10.17632/8fcntj63x6.1) and will be publicly available upon publication. Any additional information required to reproduce the data reported in this paper is available from the corresponding author upon request.

## References

[1] Schägger H, Pfeiffer K (2000). Supercomplexes in the respiratory chains of yeast and mammalian mitochondria. EMBO J..

[2] Lobo-Jarne T, Ugalde C (2018). Respiratory chain supercomplexes: structures, function and biogenesis. Semin. Cell Dev. Biol..

[3] Cruciat C-M, Brunner S, Baumann F, Neupert W, Stuart RA (2000). The cytochrome bc 1 and cytochromec oxidase complexes associate to form a single supracomplex in yeast mitochondria. J. Biol. Chem..

[4] Lopez-Fabuel I (2016). Complex I assembly into supercomplexes determines differential mitochondrial ROS production in neurons and astrocytes. Proc. Natl Acad. Sci. USA.

[5] Cogliati S (2016). Mechanism of super-assembly of respiratory complexes III and IV. Nature.

[6] Acín-Pérez R, Fernández-Silva P, Peleato ML, Pérez-Martos A, Enriquez JA (2008). Respiratory active mitochondrial supercomplexes. Mol. Cell.

[7] Milenkovic D, Blaza JN, Larsson N-G, Hirst J (2017). The enigma of the respiratory chain supercomplex. Cell Metab..

[8] Bianchi C, Genova ML, Castelli GP, Lenaz G (2004). The mitochondrial respiratory chain is partially organized in a supercomplex assembly: kinetic evidence using flux control analysis. J. Biol. Chem..

[9] Calvo, E. et al. Functional role of respiratory supercomplexes in mice: SCAF1 relevance and segmentation of the Qpool. *Sci. Adv*. 10.1126/sciadv.aba7509 (2020). 10.1126/sciadv.aba7509 PMC7314541 32637615

[10] Lapuente-Brun E (2013). Supercomplex assembly determines electron flux in the mitochondrial electron transport chain. Science.

[11] Maranzana E, Barbero G, Falasca AI, Lenaz G, Genova ML (2013). Mitochondrial respiratory supercomplex association limits production of reactive oxygen species from complex I. Antioxid. Redox Signal..

[12] Acín-Pérez R (2004). Respiratory complex iii is required to maintain complex i in mammalian mitochondria. Mol. Cell.

[13] Calvaruso MA (2012). Mitochondrial complex III stabilizes complex I in the absence of NDUFS4 to provide partial activity. Hum. Mol. Genet..

[14] Protasoni M (2020). Respiratory supercomplexes act as a platform for complex III-mediated maturation of human mitochondrial complexes I and IV. EMBO J..

[15] Schägger H (2004). Significance of respirasomes for the assembly/stability of human respiratory chain complex I. J. Biol. Chem..

[16] Jang S (2017). Elucidating mitochondrial electron transport chain supercomplexes in the heart during ischemia–reperfusion. Antioxid. Redox Signal..

[17] Rosca MG (2008). Cardiac mitochondria in heart failure: decrease in respirasomes and oxidative phosphorylation. Cardiovasc. Res..

[18] Huang Y (2015). Cardiac metabolic pathways affected in the mouse model of barth syndrome. PLoS ONE.

[19] McKenzie M, Lazarou M, Thorburn DR, Ryan MT (2006). Mitochondrial respiratory chain supercomplexes are destabilized in barth syndrome patients. J. Mol. Biol..

[20] Anwar MR, Saldana-Caboverde A, Garcia S, Diaz F (2018). The organization of mitochondrial supercomplexes is modulated by oxidative stress in vivo in mouse models of mitochondrial encephalopathy. Int. J. Mol. Sci..

[21] Arthur CR, Morton SL, Dunham LD, Keeney PM, Bennett JP (2009). Parkinson’s disease brain mitochondria have impaired respirasome assembly, age-related increases in distribution of oxidative damage to mtDNA and no differences in heteroplasmic mtDNA mutation abundance. Mol. Neurodegener..

[22] Antoun G (2015). Impaired mitochondrial oxidative phosphorylation and supercomplex assembly in rectus abdominis muscle of diabetic obese individuals. Diabetologia.

[23] Gómez LA, Monette JS, Chavez JD, Maier CS, Hagen TM (2009). Supercomplexes of the mitochondrial electron transport chain decline in the aging rat heart. Arch. Biochem. Biophys..

[24] Greggio C (2017). Enhanced respiratory chain supercomplex formation in response to exercise in human skeletal muscle. Cell Metab..

[25] García-Poyatos C (2020). Scaf1 promotes respiratory supercomplexes and metabolic efficiency in zebrafish. EMBO Rep..

[26] Williams, E. G. et al. Systems proteomics of liver mitochondria function. *Science*10.1126/science.aad0189 (2016). 10.1126/science.aad0189 PMC10859670 27284200

[27] Balsa E (2019). ER and nutrient stress promote assembly of respiratory chain supercomplexes through the PERK–eIF2α axis. Mol. Cell.

[28] Zhang K (2016). COX7AR is a stress-inducible mitochondrial COX subunit that promotes breast cancer malignancy. Sci. Rep..

[29] Hollinshead KER (2020). Respiratory supercomplexes promote mitochondrial efficiency and growth in severely hypoxic pancreatic cancer. Cell Rep..

[30] Lobo-Jarne T (2018). Human COX7A2L regulates complex iii biogenesis and promotes supercomplex organization remodeling without affecting mitochondrial bioenergetics. Cell Rep..

[31] Pérez-Pérez R (2016). COX7A2L is a mitochondrial complex III binding protein that stabilizes the III2+IV supercomplex without affecting respirasome formation. Cell Rep..

[32] Mourier A, Matic S, Ruzzenente B, Larsson N-G, Milenkovic D (2014). The respiratory chain supercomplex organization is independent of COX7a2l isoforms. Cell Metab..

[33] Li H (2019). Identifying gene function and module connections by the integration of multispecies expression compendia. Genome Res..

[34] Lonsdale J (2013). The Genotype-Tissue Expression (GTEx) project. Nat. Genet..

[35] Aguet F (2017). Genetic effects on gene expression across human tissues. Nature.

[36] THE GTEX CONSORTIUM. The GTEx Consortium atlas of genetic regulatory effects across human tissues. Science 369, 1318–1330 (2020). 10.1126/science.aaz1776 PMC7737656 32913098

[37] Auton A (2015). A global reference for human genetic variation. Nature.

[38] Lek M (2016). Analysis of protein-coding genetic variation in 60,706 humans. Nature.

[39] Mochizuki Y (2018). Combinatorial CRISPR/Cas9 approach to elucidate a far-upstream enhancer complex for tissue-specific Sox9 expression. Dev. Cell.

[40] Dominguez D (2018). Sequence, structure, and context preferences of human RNA binding proteins. Mol. Cell.

[41] Hemani G (2018). The MR-Base platform supports systematic causal inference across the human phenome. eLife.

[42] Bycroft C (2018). The UK Biobank resource with deep phenotyping and genomic data. Nature.

[43] Davies, N. M., Holmes, M. V. & Smith, G. D. Reading Mendelian randomisation studies: a guide, glossary, and checklist for clinicians. *BMJ*10.1136/bmj.k601 (2018). 10.1136/bmj.k601 PMC6041728 30002074

[44] Teumer, A. Common methods for performing Mendelian randomization. *Front. Cardiovasc. Med*. 10.3389/fcvm.2018.00051 (2018). 10.3389/fcvm.2018.00051 PMC5985452 29892602

[45] Doherty A (2018). GWAS identifies 14 loci for device-measured physical activity and sleep duration. Nat. Commun..

[46] Krokstad S (2013). Cohort profile: The HUNT study, Norway. Int. J. Epidemiol..

[47] Loe H, Steinshamn S, Wisløff U (2014). Cardio-respiratory reference data in 4631 healthy men and women 20-90 years: the HUNT 3 fitness study. PLoS ONE.

[48] Bye, A. et al. Identification of novel genetic variants associated with cardiorespiratory fitness. *Prog. Cardiovasc. Dis*. 10.1016/j.pcad.2020.02.001 (2020). 10.1016/j.pcad.2020.02.001 32035127

[49] Zeiher J (2019). Correlates and determinants of cardiorespiratory fitness in adults: a systematic review. Sports Med. Open.

[50] Eriksson, J. G. et al. Prenatal and childhood growth and physical performance in old age—findings from the Helsinki Birth Cohort Study 1934–1944. *Age*10.1007/s11357-015-9846-1 (2015). 10.1007/s11357-015-9846-1 PMC5005845 26499818

[51] Oja P, Laukkanen R, Pasanen M, Tyry T, Vuori I (1991). A 2-km walking test for assessing the cardiorespiratory fitness of healthy adults. Int. J. Sports Med..

[52] Salonen MK (2011). Developmental origins of physical fitness: the Helsinki Birth Cohort study. PLoS ONE.

[53] Laukkanen R, Oja P, Pasanen M, Vuori I (1992). Validity of a two kilometre walking test for estimating maximal aerobic power in overweight adults. Int. J. Obes. Relat. Metab. Disord..

[54] Rance M (2005). Validity of a V·O_2_max prediction equation of the 2-km walk test in female seniors. Int. J. Sports Med..

[55] Andreux PA (2012). Systems genetics of metabolism: the use of the BXD murine reference panel for multiscalar integration of traits. Cell.

[56] Jha P (2018). Genetic regulation of plasma lipid species and their association with metabolic phenotypes. Cell Syst..

[57] Wu Y (2014). Multilayered genetic and omics dissection of mitochondrial activity in a mouse reference population. Cell.

[58] Huang T-T (2006). Genetic modifiers of the phenotype of mice deficient in mitochondrial superoxide dismutase. Hum. Mol. Genet..

[59] Toye AA (2005). A genetic and physiological study of impaired glucose homeostasis control in C57BL/6J mice. Diabetologia.

[60] Marcuello A (2005). Skeletal muscle mitochondrial DNA content in exercising humans. J. Appl. Physiol..

[61] Puente-Maestu L (2011). Effects of exercise on mitochondrial DNA content in skeletal muscle of patients with COPD. Thorax.

[62] Althoff T, Mills DJ, Popot J-L, Kühlbrandt W (2011). Arrangement of electron transport chain components in bovine mitochondrial supercomplex I1III2IV1. EMBO J..

[63] Blaza JN, Serreli R, Jones AJY, Mohammed K, Hirst J (2014). Kinetic evidence against partitioning of the ubiquinone pool and the catalytic relevance of respiratory-chain supercomplexes. Proc. Natl Acad. Sci. USA.

[64] Fedor JG, Hirst J (2018). Mitochondrial supercomplexes do not enhance catalysis by quinone channeling. Cell Metab..

[65] Vercellino I, Sazanov LA (2021). Structure and assembly of the mammalian mitochondrial supercomplex CIII2CIV. Nature.

[66] Letts JA, Fiedorczuk K, Sazanov LA (2016). The architecture of respiratory supercomplexes. Nature.

[67] Gu J (2016). The architecture of the mammalian respirasome. Nature.

[68] Wu M, Gu J, Guo R, Huang Y, Yang M (2016). Structure of mammalian respiratory supercomplex I1III2IV1. Cell.

[69] Gonzalez-Franquesa A (2021). Mass-spectrometry-based proteomics reveals mitochondrial supercomplexome plasticity. Cell Rep..

[70] Granata C (2021). High-intensity training induces non-stoichiometric changes in the mitochondrial proteome of human skeletal muscle without reorganisation of respiratory chain content. Nat. Commun..

[71] Di Meo S, Venditti P (2001). Mitochondria in exercise-induced oxidative stress. Biol. Signals Recept..

[72] Poulsen HE, Loft S, Vistisen K (1996). Extreme exercise and oxidative DNA modification. J. Sports Sci..

[73] Ali AT (2019). Nuclear genetic regulation of the human mitochondrial transcriptome. eLife.

[74] Kim S, Myers L, Ravussin E, Cherry KE, Jazwinski SM (2016). Single nucleotide polymorphisms linked to mitochondrial uncoupling protein genes UCP2 and UCP3 affect mitochondrial metabolism and healthy aging in female nonagenarians. Biogerontology.

[75] Harvey NR (2020). Investigating the influence of mtDNA and nuclear encoded mitochondrial variants on high intensity interval training outcomes. Sci. Rep..

[76] Eynon N, Morán M, Birk R, Lucia A (2011). The champions’ mitochondria: is it genetically determined? A review on mitochondrial DNA and elite athletic performance. Physiol. Genomics.

[77] Papadimitriou ID (2019). A “human knockout” model to investigate the influence of the α-actinin-3 protein on exercise-induced mitochondrial adaptations. Sci. Rep..

[78] Myers J, Kokkinos P, Nyelin E (2019). Physical activity, cardiorespiratory fitness, and the metabolic syndrome. Nutrients.

[79] Al-Mallah MH, Sakr S, Al-Qunaibet A (2018). Cardiorespiratory fitness and cardiovascular disease prevention: an update. Curr. Atheroscler. Rep..

[80] Jantunen H (2017). Objectively measured physical activity and physical performance in old age. Age Ageing.

[81] Marchini J, Howie B (2010). Genotype imputation for genome-wide association studies. Nat. Rev. Genet..

[82] Moses EK (2008). Genetic association of preeclampsia to the inflammatory response gene SEPS1. Am. J. Obstet. Gynecol..

[83] Nielsen JB (2018). Biobank-driven genomic discovery yields new insight into atrial fibrillation biology. Nat. Genet..

[84] Kent WJ (2002). BLAT—the BLAST-like alignment tool. Genome Res..

[85] Purcell S (2007). PLINK: a tool set for whole-genome association and population-based linkage analyses. Am. J. Hum. Genet..

[86] Loh P-R (2016). Reference-based phasing using the Haplotype Reference Consortium panel. Nat. Genet..

[87] Das S (2016). Next-generation genotype imputation service and methods. Nat. Genet..

[88] McCarthy S (2016). A reference panel of 64,976 haplotypes for genotype imputation. Nat. Genet..

[89] Manichaikul A (2010). Robust relationship inference in genome-wide association studies. Bioinformatics.

[90] Abraham KJ, Diaz C (2014). Identifying large sets of unrelated individuals and unrelated markers. Source Code Biol. Med..

[91] Loh P-R (2015). Efficient Bayesian mixed-model analysis increases association power in large cohorts. Nat. Genet..

[92] Marees, A. T. et al. A tutorial on conducting genome‐wide association studies: quality control and statistical analysis. *Int. J. Methods Psychiatr. Res*. 10.1002/mpr.1608 (2018). 10.1002/mpr.1608 PMC6001694 29484742

[93] Ritchie ME (2015). limma powers differential expression analyses for RNA-sequencing and microarray studies. Nucleic Acids Res..

[94] Kamat MA (2019). PhenoScanner V2: an expanded tool for searching human genotype–phenotype associations. Bioinformatics.

[95] Jha P, Wang X, Auwerx J (2016). Analysis of mitochondrial respiratory chain supercomplexes using blue native polyacrylamide gel electrophoresis (BN-PAGE). Curr. Protoc. Mouse Biol..

[96] Reynaud-Dulaurier R (2020). Gene replacement therapy provides benefit in an adult mouse model of Leigh syndrome. Brain.

[97] Quiros PM, Goyal A, Jha P, Auwerx J (2017). Analysis of mtDNA/nDNA ratio in mice. Curr. Protoc. Mouse Biol..

[98] Wohlwend, M. et al. The exercise-induced long noncoding RNA CYTOR promotes fast-twitch myogenesis in aging. *Sci. Transl. Med*. 10.1126/scitranslmed.abc7367 (2021). 10.1126/scitranslmed.abc7367 34878822

[99] Ratnadiwakara, M. & Änkö, M.-L. mRNA stability assay using transcription inhibition by actinomycin D in mouse pluripotent stem cells. *Bio. Protoc*. 10.21769/bioprotoc.3072 (2018). 10.21769/BioProtoc.3072 PMC8342049 34532533

