# Supplementary material for: COX7A2L genetic variants determine cardiorespiratory fitness in mice and human
Source: Nat Metab. 2022 Oct 17;4(10):1336–51. doi: 10.1038/s42255-022-00655-0 (PMC9584823; doi:10.1038/s42255-022-00655-0)
Supplement: Supplementary file 1 — Reporting Summary [file 42255_2022_655_MOESM1_ESM.pdf]

nature portfolio

Corresponding author(s): Johan Auwerx
Last updated by author(s): Aug 28, 2022

Reporting Summary

Nature Portfolio wishes to improve the reproducibility of the work that we publish. This form provides structure for consistency and transparency in reporting. For further information on Nature Portfolio policies, see our Editorial Policies and the Editorial Policy Checklist.

Statistics

For all statistical analyses, confirm that the following items are present in the figure legend, table legend, main text, or Methods section.

n/a	Confirmed	
☐	☒	The exact sample size (*n*) for each experimental group/condition, given as a discrete number and unit of measurement
☐	☒	A statement on whether measurements were taken from distinct samples or whether the same sample was measured repeatedly
☐	☒	The statistical test(s) used AND whether they are one- or two-sided<br>*Only common tests should be described solely by name; describe more complex techniques in the Methods section.*
☐	☒	A description of all covariates tested
☐	☒	A description of any assumptions or corrections, such as tests of normality and adjustment for multiple comparisons
☐	☒	A full description of the statistical parameters including central tendency (e.g. means) or other basic estimates (e.g. regression coefficient) AND variation (e.g. standard deviation) or associated estimates of uncertainty (e.g. confidence intervals)
☐	☒	For null hypothesis testing, the test statistic (e.g. *F*, *t*, *r*) with confidence intervals, effect sizes, degrees of freedom and *P* value noted<br>*Give P values as exact values whenever suitable.*
☒	☐	For Bayesian analysis, information on the choice of priors and Markov chain Monte Carlo settings
☒	☐	For hierarchical and complex designs, identification of the appropriate level for tests and full reporting of outcomes
☐	☒	Estimates of effect sizes (e.g. Cohen's *d*, Pearson's *r*), indicating how they were calculated

Our web collection on statistics for biologists contains articles on many of the points above.

Software and code

Policy information about availability of computer code

Data collection	qPCR reactions were performed using the Light-Cycler system (Roche Applied Science). Victor X4 plate reader (Perkin Elmer) was used for assays requiring luminescence quantifications. Cellular respiration was measured using Seahorse analyser (XF96, Agilent Technologies). Western blots were imaged using Fusion FX6 imaging system (Vilber). In the HUNT study oxygen uptake kinetics were measured directly by a portable mixing chamber gas-analyzer (Cortex MetaMax II, Cortex, Leipzig, Germany). In mice, VO2max was measured with a calorimetric treadmill (Columbus instruments, Columbus, OH, USA). Energy expenditure, VO2, VCO2, food intake, cage and wheel activity were measured using the Promethion system (Sable Systems International)
Data analysis	GraphPad Prism 7 (GraphPad Software, Inc.), R version 3.5.2., IMPUTE2 software, PLINK v1.90, Eagle2 v2.3.8, Minimac3 v2.0.1. Essential scripts used in this study were deposited at Mendeley Data (doi: 10.17632/8fcntj63x6.1)

For manuscripts utilizing custom algorithms or software that are central to the research but not yet described in published literature, software must be made available to editors and reviewers. We strongly encourage code deposition in a community repository (e.g. GitHub). See the Nature Portfolio guidelines for submitting code & software for further information.

Data

Policy information about availability of data

All manuscripts must include a data availability statement. This statement should provide the following information, where applicable:

- Accession codes, unique identifiers, or web links for publicly available datasets
- A description of any restrictions on data availability
- For clinical datasets or third party data, please ensure that the statement adheres to our policy

The data generated in this study are available with the published manuscript and/or were deposited at Mendeley Data (doi: 10.17632/8fcntj63x6.1) and will be publicly available upon publication. The GTEx gene expression and eQTL data are publicly available at http://www.gtexportal.org/home/. Ontology terms and pathways correlated to COX7A2L can be found through the publicly available GeneBridge tool (https://systems-genetics.org). Genotype frequencies data from the 1000 genomes project phase 3 and gnomAD are publicly available at https://www.ensembl.org/. The ieu gwas database is publicly available at https://gwas.mrcieu.ac.uk/datasets/.

Field-specific reporting

Please select the one below that is the best fit for your research. If you are not sure, read the appropriate sections before making your selection.

☒ Life sciences ☐ Behavioural & social sciences ☐ Ecological, evolutionary & environmental sciences

For a reference copy of the document with all sections, see nature.com/documents/nr-reporting-summary-flat.pdf

Life sciences study design

All studies must disclose on these points even when the disclosure is negative.

Sample size	No statistical method was used to determine sample size. Sample sizes were chosen based on studies with similar experimental design and on the known variability of the assay. For example, VO2max and energy expenditure measurements in the mice have high variability, therefore groups of 15-19 mice were analysed. The number of myoblast lines was chosen based on the available homozygous lines out of a library of 15 different lines.
Data exclusions	Mice that showed signs of disease, predefined by the animal authorizations, were euthanized and removed from the study. In the Promethion experiment mice that did not spontaneously run on the running wheel were excluded from the analysis
Replication	The experiments were repeated at least twice, and replication attempts were successful. All the replicates represent biological replicates. The mice used for experiments were from multiple different litters.
Randomization	For all the in vivo and in vitro experiments, groups were randomly assigned. The human genetic association analysis were adjusted for sex and age.
Blinding	Scientists performing the mouse phenotyping experiments were blinded to the experimental groups. The in vitro experiments were performed by a single investigator and blinding was not possible

Reporting for specific materials, systems and methods

We require information from authors about some types of materials, experimental systems and methods used in many studies. Here, indicate whether each material, system or method listed is relevant to your study. If you are not sure if a list item applies to your research, read the appropriate section before selecting a response.

Materials & experimental systems

n/a	Involved in the study	
☐	☒	Antibodies
☐	☒	Eukaryotic cell lines
☒	☐	Palaeontology and archaeology
☐	☒	Animals and other organisms
☐	☒	Human research participants
☒	☐	Clinical data
☒	☐	Dual use research of concern

Methods

n/a	Involved in the study	
☒	☐	ChIP-seq
☒	☐	Flow cytometry
☒	☐	MRI-based neuroimaging

Antibodies

Antibodies used	Total OXPHOS Rodent WB Antibody Cocktail, Abcam, Cat# ab110413 (1:1000)<br>Anti-NDUFS3, Abcam, Cat# ab14711 (1:2000)

Anti-UQCRC2, Abcam, Cat# ab14745 (1:10000)
Anti-MTCO1, Abcam, Cat# ab14705 (1:1000)
Anti-Cox7a2l, St John's Laboratory, STJ110597 (1:1000)
Anti-Cox7a2l, Proteintech, Cat# 11416-1-AP (1:1000)
Anti-Tfam, Abcam, Cat# ab131607 (1:1000)
Anti-VDAC1, Abcam, Cat# ab14734 (1:1000)
Anti-PGC1a, Calbiochem, Cat# ST1202 (1:1000)
Anti-Vinculin, Abcam, Cat# ab129002 (1:1000)
Anti-Sdha antibody (Abcam, ab14715, 1:1000)
Anti-HA tag, Abcam, Cat# ab9110 (5ug/sample)
IgG control, Santa Cruz Biotechnology, Cat# sc-2025 (5ug/sample)

Validation

All the antibodies were validated by the manufacturer. See more details below:

Total OXPHOS Rodent WB Antibody Cocktail, Abcam, Cat# ab110413 (1:1000)
Total OXPHOS Rodent WB Antibody Cocktail ab110413 is an optimized cocktail of high quality antibodies for analyzing relative levels of OXPHOS complexes in rat or mouse mitochondria by western blot.
This OXPHOS cocktail contains 5 mouse mAbs, one each against CI subunit NDUFB8 (ab110242), CII-30kDa (ab14714), CIII-Core protein 2 (ab14745), CIV subunit I (ab14705) and CV alpha subunit (ab14748) as an optimized premixed cocktail. The kit is suitable for Western Blotting analysis of the relative levels of the 5 OXPHOS complexes in mitochondrial preparations from mouse, rat, human, or bovine sources.

Anti-NDUFS3, Abcam, Cat# ab14711 (1:2000)
Mouse monoclonal [17D95] to NDUFS3
Suitable for: WB, Flow Cyt
Reacts with: Mouse, Rat, Cow, Human

Anti-UQCRC2, Abcam, Cat# ab14745 (1:10000)
Mouse monoclonal [13G12AF12BB11] to UQCRC2
Suitable for: Flow Cyt, IHC-P, WB
Reacts with: Human

Anti-MTCO1, Abcam, Cat# ab14705 (1:1000)
Mouse monoclonal [1D6E1A8] to MTCO1
Suitable for: ICC, IHC-P, WB, Flow Cyt
Reacts with: Mouse, Rat, Cow, Human

Anti-Cox7a2l, St John's Laboratory, STJ110597 (1:1000)
Applications:WB
Reactivity:Human, Mouse, Rat
Short Description: Rabbit polyclonal antibody anti-COX7A2L (1-114) is suitable for use in Western Blot.
Immunogen:Recombinant fusion protein containing a sequence corresponding to amino acids 1-114 of human COX7A2L (NP_004709.2).

Anti-Cox7a2l, Proteintech, Cat# 11416-1-AP (1:1000)
COX7A2L Polyclonal Antibody for IHC, WB,ELISA
KO validated

Anti-Tfam, Abcam, Cat# ab131607 (1:1000)
Rabbit polyclonal to mtTFA - Mitochondrial Marker
Suitable for: WB
Reacts with: Rat

Anti-VDAC1, Abcam, Cat# ab14734 (1:1000)
Mouse monoclonal [20B12AF2] to VDAC1/Porin + VDAC3
Suitable for: WB, ICC/IF, Flow Cyt
Reacts with: Mouse, Rat, Cow, Human

Anti-PGC1a, Calbiochem, Cat# ST1202 (1:1000)
Protein G purified mouse monclonal antibody. Recognizes the endogenous forms of PGC-1α, which includes the ~113 kDa PGC-1α protein and the ~38 kDa splice variant.
Recognizes the endogenous forms of PGC-1α, which includes the ~113 kDa PGC-1α protein and the ~38 kDa splice variant, in brown adipose tissue, liver, and kidney.
This Anti-PGC-1α Mouse mAb (4C1.3) is validated for use in Immunoblotting, Immunocytochemistry, Immunoprecipitation, Paraffin Sections for the detection of PGC-1α.
Immunogen: Mouse. a recombinant protein consisting of amino acids 1-120 of mouse PGC-1α

Anti-Vinculin, Abcam, Cat# ab129002 (1:1000)
Produced recombinantly (animal-free) for high batch-to-batch consistency and long term security of supply

Rabbit monoclonal [EPR8185] to Vinculin
Suitable for: Flow Cyt (Intra), WB, IP, ICC/IF
Reacts with: Mouse, Rat, Human

Anti-Sdha antibody (Abcam, ab14715, 1:1000)
Mouse monoclonal [2E3GC12FB2AE2] to SDHA
Suitable for: IHC-Fr, Flow Cyt, WB, ICC, IHC-P
Knockout validated
Reacts with: Mouse, Rat, Cow, Human

Anti-HA tag, Abcam, Cat# ab9110 (5ug/sample)
Rabbit polyclonal to HA tag - ChIP Grade
Suitable for: ChIP/Chip, IP, ELISA, WB, ICC/IF, Flow Cyt, ChIP
Reacts with: Species independent

IgG control, Santa Cruz Biotechnology, Cat# sc-2025 (5ug/sample)
use as negative control for Western blotting, immunoprecipitation and immunohistochemistry applications
cited 3242 times

Eukaryotic cell lines

Policy information about cell lines

Cell line source(s)	Hek293T were obtained from ATCC, Human myoblast lines were obtained from the CBC BioTec Centre de Biologie et Pathologie EST Groupement Hospitalier Est, 59 Bd Pinel, 69677 BRON cedex
Authentication	Hek293T were authenticated by STR profiling. Human myoblasts were authenticated by morphology and ability to differentiate into myotubes
Mycoplasma contamination	All cell lines tested negative for mycoplasma contamination
Commonly misidentified lines (See ICLAC register)	No commonly misidentified cell lines were used

Animals and other organisms

Policy information about studies involving animals; ARRIVE guidelines recommended for reporting animal research

Laboratory animals	For animal experiments, C57BL/6J or DBA/2J 12-15 weeks old male mice were used
Wild animals	The study did not involve wild animals
Field-collected samples	The study did not involve samples collected from the field
Ethics oversight	All animal experiments were performed according to Swiss ethical guidelines and approved by the Service de la Consommation et des Affaires Vétérinaires (SCAV) of the Canton de Vaud (license VD3419)

Note that full information on the approval of the study protocol must also be provided in the manuscript.

Human research participants

Policy information about studies involving human research participants

Population characteristics	The population characteristics of the HUNT3 and Helsinki Birth Cohort Studies were described previously (https://doi.org/10.1371/journal.pone.0113884, https://doi.org/10.1371/journal.pone.0022302)
Recruitment	For the HUNT3 Fitness Study, exclusion criteria were present or previous heart disease, stroke, angina, lung disease (asthma, chronic bronchitis, chronic obstructive pulmonary disease, and sarcoidosis), cancer, current pregnancy, orthopedic limitations and use of hypertensive medication. For a more detailed descriprion see https://doi.org/10.1371/journal.pone.0113884 and https://doi.org/10.1016/j.pcad.2020.02.001.<br>The recruitment of the Helsinki Birth Cohort Study participants was described previously (https://doi.org/10.1371/journal.pone.0022302)
Ethics oversight	The HUNT3 fitness study was approved by the Regional committee for medical research ethics (2012/1672/REK nord), the Norwegian Data Inspectorate and the National Directorate of Health, and is in compliance with the Helsinki declaration. Written informed consent was obtained from all participants.<br>The clinical Helsinki Birth Cohort study protocol was approved by the Ethics Committee of Epidemiology and Public Health of the Hospital District of Helsinki and Uusimaa. Written informed consent was obtained from each participant before any study

procedure was initiated

Note that full information on the approval of the study protocol must also be provided in the manuscript.
