# Supplementary figures and images for: COX7A2L genetic variants determine cardiorespiratory fitness in mice and human
Source: Nat Metab. 2022 Oct 17;4(10):1336–51. doi: 10.1038/s42255-022-00655-0 (PMC9584823; doi:10.1038/s42255-022-00655-0)

Source Blots Figure 3

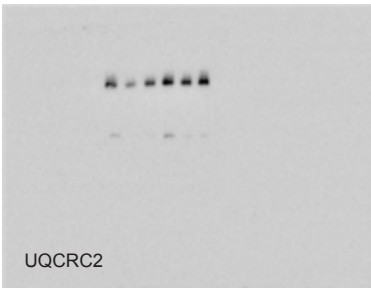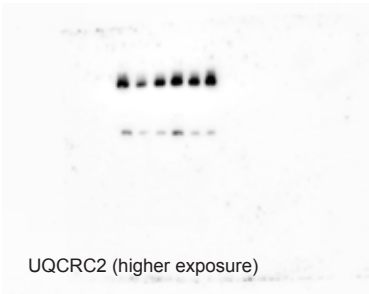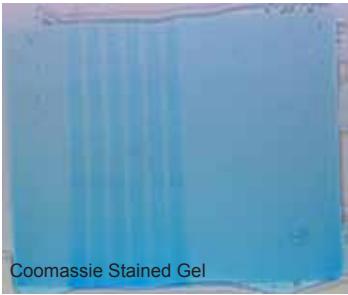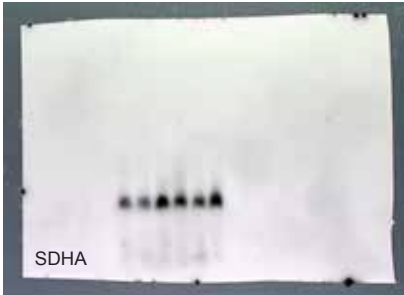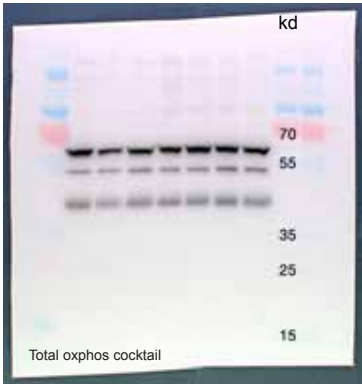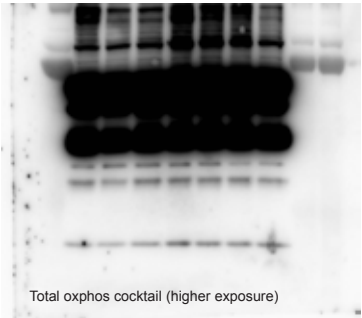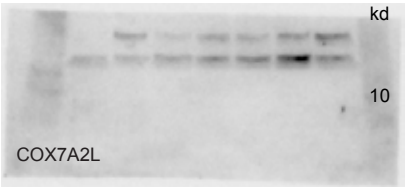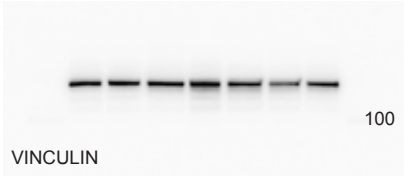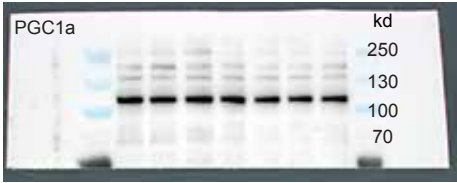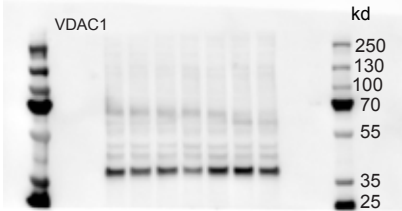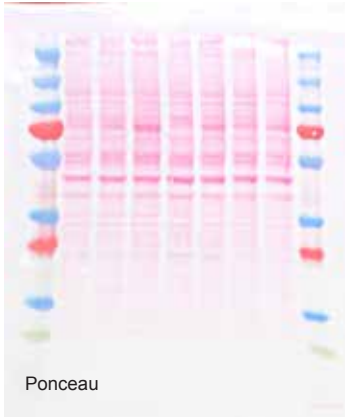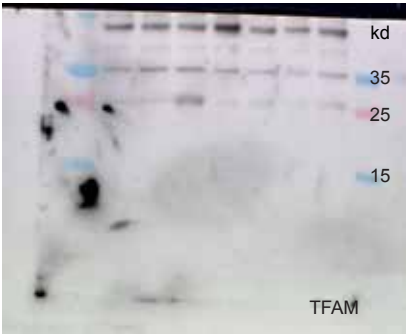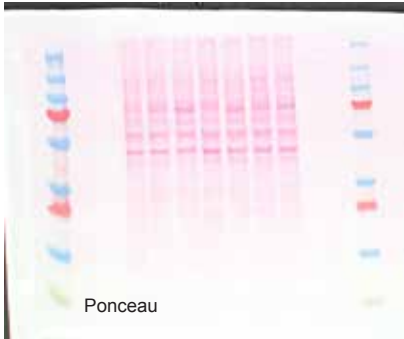

Supplement: Source Data Fig. 3 — Unprocessed western blots and/or gels. [file 42255_2022_655_MOESM5_ESM.pdf]

Source Blots Figure 6

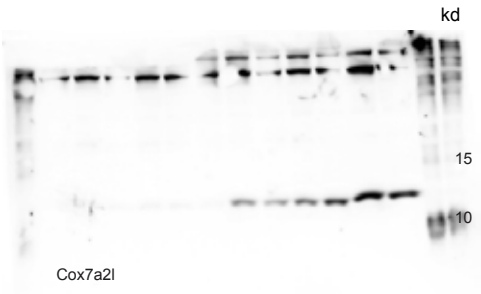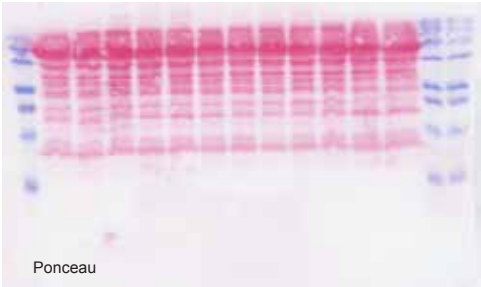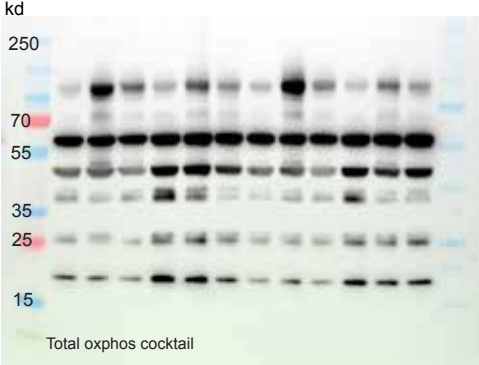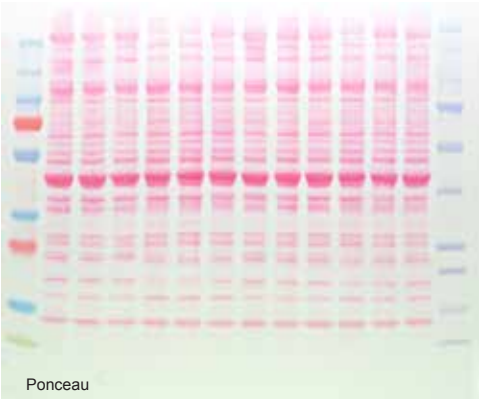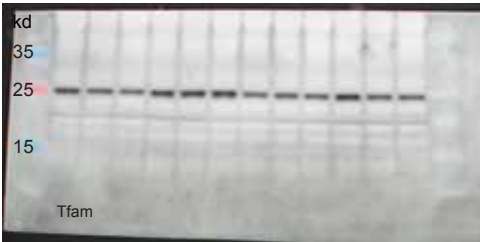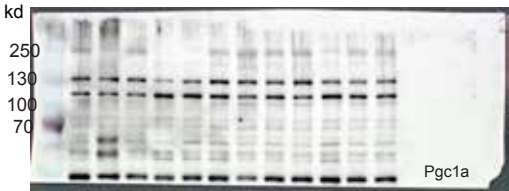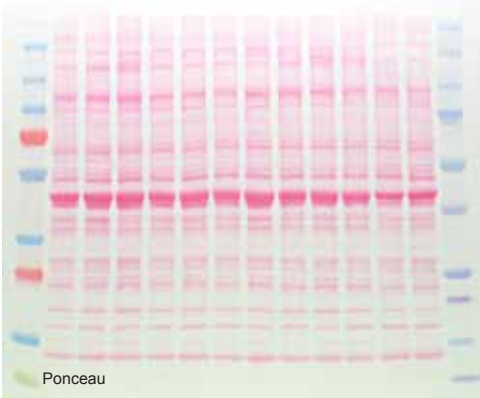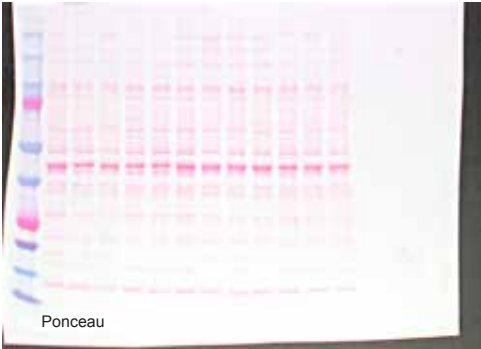

Source Blots Figure 6

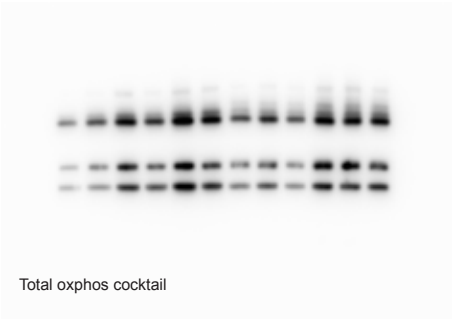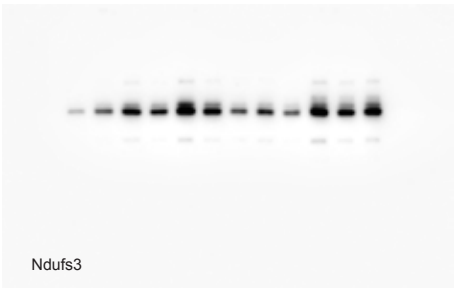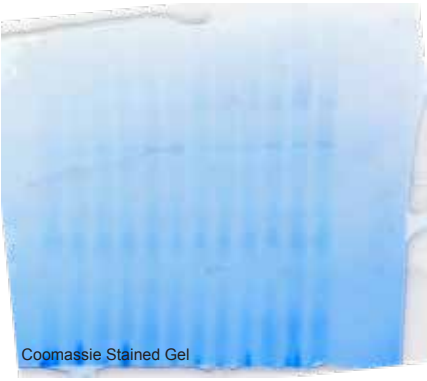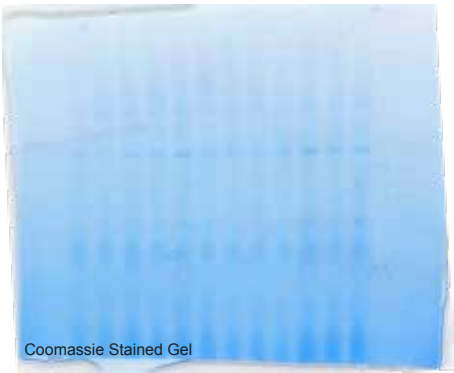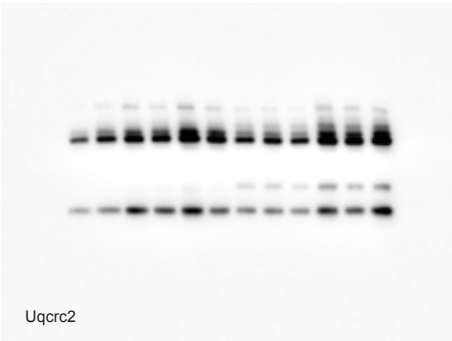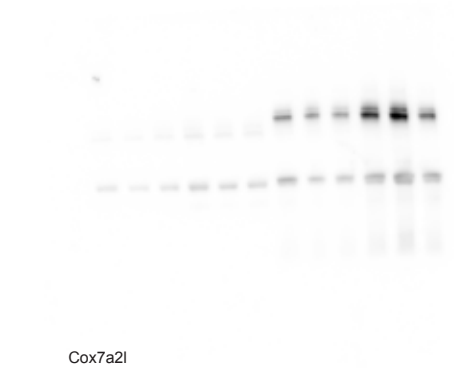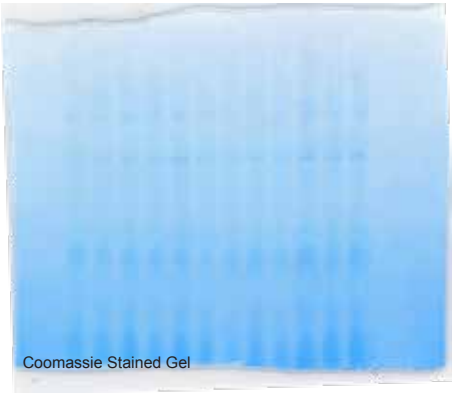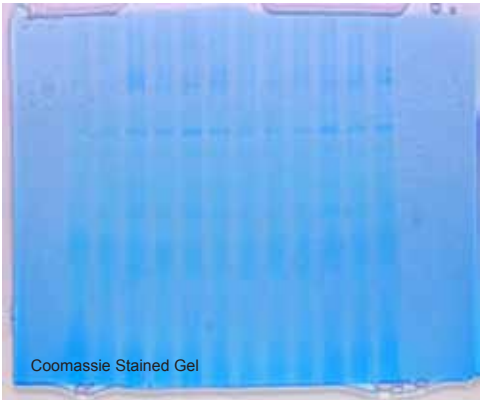

Supplement: Source Data Fig. 6 — Unprocessed western blots and/or gels. [file 42255_2022_655_MOESM8_ESM.pdf]

Source Blots Extended Data Fig. 3

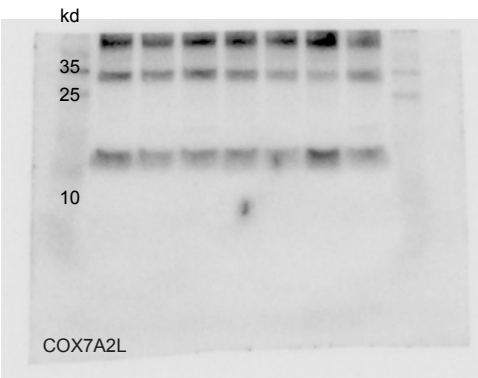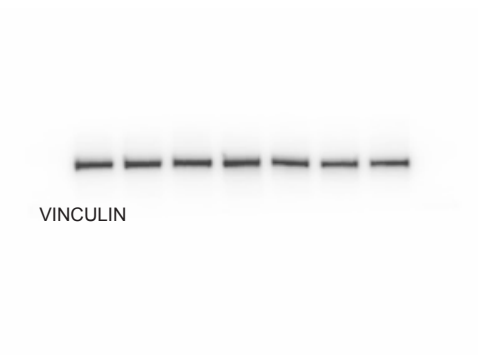

Supplement: Source Data Extended Data Fig. 3 — Unprocessed western blots and/or gels. [file 42255_2022_655_MOESM11_ESM.pdf]

Source Blots Extended Data Fig.6

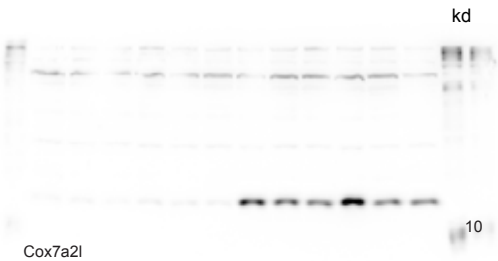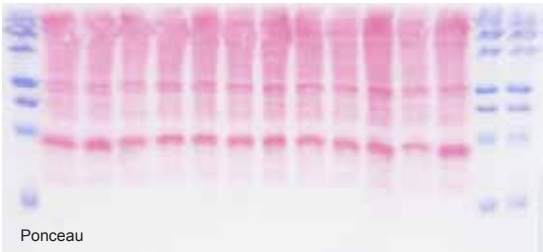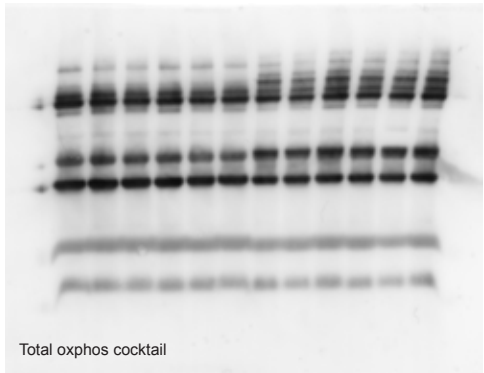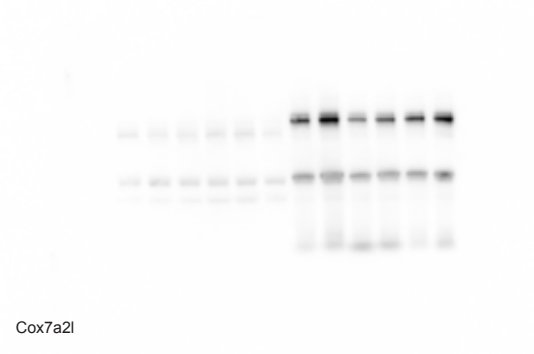

Supplement: Source Data Extended Data Fig. 6 — Unprocessed western blots and/or gels. [file 42255_2022_655_MOESM15_ESM.pdf]
